# Multimodal Large Language Models for Cystoscopic Image Interpretation and Bladder Lesion Classification: Comparative Study

**DOI:** 10.2196/87193

**Published:** 2026-01-28

**Authors:** Yung-Chi Shih, Cheng-Yang Wu, Shi-Wei Huang, Chung-You Tsai

**Affiliations:** 1 Division of Urology Department of Surgery Far Eastern Memorial Hospital New Taipei City Taiwan; 2 Department of Urology National Taiwan University Hospital, Yunlin Branch Yunlin Taiwan; 3 Department of Urology College of Medicine National Taiwan University Taipei Taiwan; 4 Department of Electrical Engineering Yuan Ze University Taoyuan Taiwan

**Keywords:** multimodal, large language model, AI, cystoscopy, diagnostic reasoning, finding description, biopsy indication, bladder tumor, artificial intelligence

## Abstract

**Background:**

Cystoscopy remains the gold standard for diagnosing bladder lesions; however, its diagnostic accuracy is operator dependent and prone to missing subtle abnormalities such as carcinoma in situ or misinterpreting mimic lesions (tumor, inflammation, or normal variants). Artificial intelligence–based image-analysis systems are emerging, yet conventional models remain limited to single tasks and cannot produce explanatory reports or articulate diagnostic reasoning. Multimodal large language models (MM-LLMs) integrate visual recognition, contextual reasoning, and language generation, offering interpretive capabilities beyond conventional artificial intelligence.

**Objective:**

This study aims to rigorously evaluate state-of-the-art MM-LLMs for cystoscopic image interpretation and lesion classification using clinician-defined stress-test datasets enriched with rare, diverse, and challenging lesions, focusing on diagnostic accuracy, reasoning quality, and clinical relevance.

**Methods:**

Four MM-LLMs (OpenAI-o3 and ChatGPT-4o [OpenAI]; Gemini 2.5 Pro and MedGemma-27B [Google]) were evaluated under blinded, randomized procedures across two tasks: (1) free-text image interpretation for anatomic site, findings, lesion reasoning, and final diagnosis (n=401) and (2) seven-class tumor-like lesion classification (n=113) within a multiple-choice framework (cystitis, polyps, papilloma, papillary urothelial carcinoma, carcinoma in situ, non-urothelial carcinoma, and none of the above). Three raters independently scored outputs using a 5-point Likert scale, and classification metrics (accuracy, sensitivity, specificity, Youden J index (Youden J), and Matthews correlation coefficient [MCC]) were calculated for lesion detection, biopsy indication, and malignancy endpoints. For optimization, model performance was compared between zero-shot and text-based in-context learning prompts that were prefixed with brief descriptions of tumor features.

**Results:**

The 401-image test set spanned 40 subcategories, with 322 (80.3%) containing abnormal findings in the image interpretation task. OpenAI-o3 demonstrated strong reasoning, with high satisfaction for anatomy (339/401, 84.5%) and findings (305/401, 76%), but lower satisfaction for lesion reasoning (211/401, 52.5%) and final diagnosis (193/401, 48.2%), indicating increasing difficulty with higher-order synthesis. Mean Likert score differences (OpenAI-o3 minus Gemini 2.5 Pro) were +0.27 for findings (adjusted *P* value: q=0.002), +0.24 for lesion reasoning (q=0.047), and +0.19 for final diagnosis. For clinically relevant endpoints in the full set, OpenAI-o3 achieved the most balanced performance, with lesion detection accuracy of 88.3%, sensitivity of 92%, specificity of 73.1%, Youden J of 0.650, and MCC of 0.635. In 7-class tumor-like lesion classification, OpenAI-o3 achieved accuracies of 73.5% for biopsy indication and 62.8% for malignancy, with a balanced sensitivity-specificity trade-off, outperforming other models. Notably, OpenAI-o3 performed best on prevalent malignant lesions. ChatGPT-4o and Gemini 2.5 Pro showed high sensitivity but low specificity, whereas MedGemma-27B underperformed. In-context learning improved OpenAI-o3 microaverage accuracy (40.7%→46.0%; MCC 0.311→0.370) but yielded only slight specificity gains and minimal accuracy change in other models, likely constrained by the absence of paired image-text context.

**Conclusions:**

MM-LLMs demonstrate meaningful assistive potential in generating interpretable cystoscopy free-text rationales and supporting biopsy triage and training. However, performance in difficult differential diagnoses remains modest and requires further optimization before safe clinical integration.

## Introduction

Cystoscopy is one of the most frequently performed procedures in urology [1]. Its effectiveness heavily depends on the urologist’s experience, attention to detail, and interpretive skill, making it both technically and diagnostically challenging [2]. Interobserver variability is common, and lesion characterization (tumor vs inflammation vs normal variant) is not always straightforward, often requiring clinical correlation. Bladder cancer, the ninth most common cancer globally [3], relies heavily on cystoscopy as the cornerstone for diagnosis, treatment, and surveillance. However, studies report false-negative rates ranging from 10%-40%, with white-light cystoscopy missing up to one-third of carcinoma in situ (CIS) cases and frequently overlooking small tumors [4]. Accordingly, cystoscopic interpretation is a nuanced clinical process.

Artificial intelligence (AI)-assisted cystoscopic diagnosis and decision-making can be decomposed into distinct tasks: lesion detection (present vs absent), lesion classification, margin segmentation, descriptive reporting, biopsy triage, final diagnosis, and ultimately full report generation. Each task places different demands on algorithms, ranging from visual localization to semantic reasoning and clinical judgment. Previous work in cystoscopy has predominantly framed the problem as image classification or segmentation [5-9], often using specialized vision pipelines that localize or outline lesions but provide limited clinical context and have uncertain generalizability across morphology-diverse appearances.

Evidence from other endoscopic domains provides a useful benchmark. Task-tuned computer-aided detection systems in colonoscopy, for example, improve clinically meaningful endpoints such as polyp or adenoma detection in randomized and real-world settings; however, these gains are achieved by narrowly optimized, single-purpose models rather than by systems capable of broader interpretive reasoning [10-14].

Against this background, multimodal large language models (MM-LLMs) hold substantial potential [15]. By jointly processing images and text, MM-LLMs can, in principle, “see and say”: integrate visual features with medical knowledge, generate free-text rationales, and condition decisions on clinical context [16]. Early reports suggest encouraging aggregate performance, but also reveal marked variability across lesions and tasks, indicating a role as assistive rather than autonomous readers at present [17].

Key gaps remain. First, it is unclear how state-of-the-art (SOTA) MM-LLMs perform on morphology-diverse, clinically difficult cystoscopic images curated as a stress test by domain experts. Second, the alignment between their free-text reasoning and expert judgment has not been systematically examined. Third, the practical utility of in-context learning (ICL) in cystoscopy—without task-specific fine-tuning—remains uncertain [18].

To address these gaps, our goal was to characterize the current capabilities and limitations of MM-LLMs in cystoscopic interpretation and to outline directions for model strengthening and additional adaptations required for safe clinical adoption.

## Methods

### Overview

**Figure 1:**
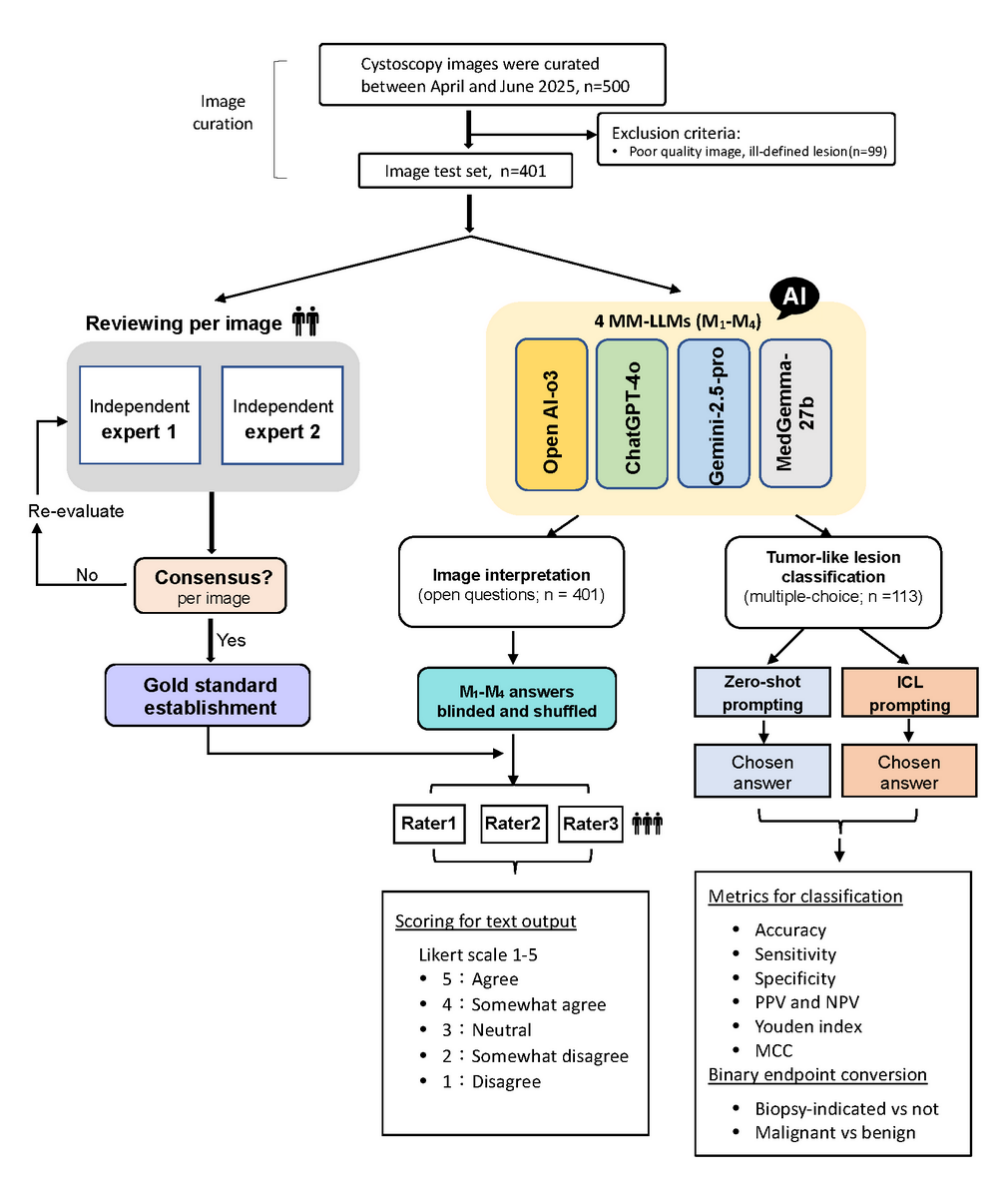
Study flow diagram. Evaluation pipeline for cystoscopic image interpretation and tumor-like lesion classification using 4 multimodal large language models (MM-LLMs). ICL: in-context learning; MCC: Matthews correlation coefficient; NPV: negative predictive value; PPV: positive predictive value.

Building on this objective, we (1) constructed a clinician-defined stress test that reflects real-world interpretive difficulty and spans benign and malignant lesions; (2) implemented a rater-blinded, model-anonymized evaluation across 2 complementary tasks—free-text image interpretation (4 open-ended questions plus a binary lesion detection query) and structured 7-class lesion classification; (3) mapped model outputs to clinically actionable binary endpoints (biopsy indication and malignancy); and (4) quantified the incremental benefit of ICL over zero-shot prompting. The overall study workflow is provided in Figure 1.

### Curation of a Diverse Image Test Set

#### Inclusion Criteria and Diverse Lesion Coverage

We evaluated vision-enabled LLMs for cystoscopic image interpretation and tumor-like lesion classification. To stress-test model generalization, the test set was curated to maximize the diversity of morphological patterns rather than mirror clinical prevalence. Images were included only if they mapped to a prespecified schema of lower urinary tract presentations—normal anatomy, intraluminal nonmucosal conditions, and focal mucosal lesions—with finer-grained sublabels (eg, verumontanum, trabeculation, and papillary urothelial carcinoma [pUC]). To extend beyond routine cases, we deliberately sampled uncommon entities encountered in practice, including endometriosis, miscellaneous deposits, fistulas, and erosion-related changes. This approach yielded a corpus with broad lesion coverage suitable for rigorous stress-testing cystoscopic interpretation.

#### Sources and Image Preprocessing

Images were curated between April and June 2025 from five sources: (1) an industry archive of intra-operative images captured on Karl-Storz endoscopes, (2) reference atlases (eg, Springer’s *Diagnostic Cystoscopy* [19] and other urologic textbooks), (3) deidentified cystoscopy images obtained from websites, (4) open-access repositories accompanying PubMed-indexed papers and public datasets, and (5) Creative Commons–licensed surgical or teaching videos hosted on YouTube (Google). A total of 500 images were curated, and 99 were excluded due to poor image quality or an ill-defined lesion. The distribution of image sources and the memorization-test results, which were used to evaluate potential data-leakage risk from overlap with the 4 models’ pretraining corpora, are summarized in Table S1 in Multimedia Appendix 1. Raw images were center-cropped to a square aspect ratio, resized to 800×800 pixels, and saved as JPEG files. This standardized pipeline harmonized the field of view and resolution across heterogeneous sources and ensured uniform inputs for all downstream model evaluations.

### Multimodal LLMs

The evaluated MM-LLMs comprised 3 general-purpose models and 1 open-weight, medical-specific baseline. Two reasoning-optimized models—OpenAI-o3 [20] and Google Gemini 2.5 Pro [21]—were selected for their native image processing capabilities and emphasis on multistep reasoning. These represent the SOTA reasoning MM-LLMs available before July 2025. ChatGPT-4o (OpenAI) was included as a general-purpose, nonreasoning MM-LLM optimized for everyday assistance and among the earliest widely deployed models capable of accepting image input. MedGemma-27B (Google) [22] was intentionally included as a local and open-weight baseline—an open-source medical model (~27B parameters) suited for on-premises deployment and potential clinical fine-tuning. Because its parameter count and training budget are substantially smaller than the proprietary models (undisclosed), MedGemma-27B serves as a baseline rather than a capacity-matched comparator. Collectively, these systems span closed-source production platforms and an open-weight medical baseline, enabling a balanced, transparent comparison.

### Study Design

#### Establishment of the Gold Standard

The reference standard diagnoses were determined through a multiphase, consensus-based process. Two urological experts, each with more than 25 years of clinical experience, independently reviewed all cystoscopic images, blinded to each other’s assessments. The initial interexpert agreement was satisfactory (Cohen κ=0.81). In cases of disagreement, a consensus meeting was convened to establish a single unified diagnosis, integrating both normal anatomical features and pathological findings.

#### Image Interpretation and Lesion Classification Tasks

For a comprehensive evaluation, we designed 2 complementary tasks that reflect distinct components of diagnostic reasoning. The first, the image interpretation task, evaluated each model’s capacity for domain-specific interpretation, logical reasoning, descriptive accuracy, and clinical judgment. The second, the lesion classification task, assessed the model’s discriminative performance in differential diagnosis. Together, these 2 tasks provided a systematic assessment of MM-LLMs in both free-form interpretation and constrained classification settings, thereby capturing complementary dimensions of clinical decision-making.

#### Image Interpretation Task

The task used a structured, stepwise, open-ended question format. Each model was primed with a role-based instruction (“Suppose you are a urologist”) and prompted with 4 sequential open-ended questions addressing anatomical site (Q1), findings (Q2), lesion reasoning (Q4), and final diagnosis (Q5). Q3 was not an open-ended interpretation item; it was a binary lesion-detection query (present or absent) embedded in the Q1→Q5 chain-of-thought to assess abnormal-versus-normal detection and was automatically graded against the gold standard. Free-text outputs for Q1, Q2, Q4, and Q5 were independently assessed by 3 raters (urology residents with 2-5 years of cystoscopy experience) using a 5-point Likert scale (1=disagree, 2=somewhat disagree, 3=neutral, 4=somewhat agree, and 5=agree).

#### Blinded and Randomized Evaluation Procedures

A dedicated evaluation software was developed to ensure complete rater blinding and randomization of both image presentation and model output order (Figure S1 in Multimedia Appendix 1).

- Image-level randomization: the display order of images was randomized once and shared across all raters. Each evaluation screen presented only 1 cystoscopic image at a time.
- Structured display: the upper panel displayed the image and its gold-standard answers to 5 reference questions (4 open-ended and 1 binary detection). The lower panel simultaneously presented anonymized text responses from the 4 MM-LLMs.
- Model-level randomization and anonymization: for each image, the order of model outputs was independently shuffled to minimize position bias. Model identities were fully anonymized to raters.
- Scoring process: raters independently scored the free-text responses using the 5-point Likert scale. The binary lesion-detection item (Q3) was automatically graded against the reference standard and was not rated by humans.

#### Lesion Classification Task

The lesion classification task was conducted to evaluate the models’ discriminative capacity. It simulates a clinical scenario in which a urologist has already identified a tumor-like lesion and requires a differential diagnosis. A subset of tumor-like lesion images was used for this analysis. The task involved a 7-class multiple-choice framework comprising cystitis, polyps, papilloma, pUC, CIS, nonurothelial carcinoma (non-U Ca), and none of the above (NOTA). Models were tested under 2 prompting strategies: zero-shot prompting and ICL. In the ICL condition, a text-based description of tumor-related features was incorporated into the prompt. This task aimed to assess each model’s discriminative performance, adaptability to structured clinical classification, and robustness across prompting paradigms.

A subset of tumor-like lesion images was used for this task. The 7-class classification included cystitis, polyps, papilloma, pUC, CIS, non-U Ca, and NOTA. Models were tested under 2 settings—zero-shot prompting and ICL with added tumor-feature descriptions—to assess discriminative performance.

#### Clinically Relevant Binary Endpoint Conversion

To mirror real-world cystoscopic decision-making when tumor-like lesions are encountered, the 7-class classification task was collapsed into 2 clinically oriented binary endpoints. The first, the biopsy-indication endpoint, represented immediate clinical decision-making: pUC, CIS, non-U Ca, papilloma, and polyps were labeled as “biopsy indicated,” whereas cystitis and NOTA were labeled as “biopsy not indicated.” The second, the malignancy endpoint, classifies pUC, CIS, and non-U Ca as malignant, and cystitis, polyps, papilloma, and NOTA as nonmalignant. This mapping preserved the full 7-class framework for granular analysis while providing pragmatic outcomes aligned with bedside triage. Notably, papilloma—though histologically benign—was categorized as “biopsy indicated” to reflect the routine need for histologic confirmation.

### Prompt Design

The complete and exact prompt designs are detailed in the Multimedia Appendix 1.

#### Prompt Design With Open-Ended Questions for Image Interpretation

We used a role-based, zero-shot prompt tailored to cystoscopy. The prompt primed domain reasoning (“Suppose you are a urologist”) and briefly contextualized the procedure, followed by stepwise instructions to encourage explicit intermediate reasoning. The query comprised five domains: (1) anatomic site (free text), (2) endoscopic findings (free text), (3) presence or absence of a pathological lesion (binary), (4) lesion diagnostic reasoning and justification if present (free text), and (5) final diagnosis (free text).

#### Prompt Design for Tumor-Like Lesion Classification Task With Multiple-Choice Diagnostic Framework

We compared 2 prompting strategies for cystoscopic diagnosis of tumor-like lesions: zero-shot and ICL. Both adopted a role-based instruction (“Suppose you are a urologist”). The zero-shot prompt presented a single forced-choice 7-class label set. In contrast, the ICL prompt prefixed brief text-based descriptions of the 7 lesion classes before the same multiple-choice query. Models were instructed to provide the best diagnosis from the given options and include a concise rationale grounded in endoscopic morphology.

### Outcome Measures and Statistical Analysis

For each image-question-answer instance, the 3 raters’ Likert-scale ratings were averaged to obtain a single consensus score. The distribution of these scores across the test set was summarized using the mean and SD to describe the central tendency and variability of model performance. To compare performance among models, pairwise differences in scores were analyzed using paired *t* tests. Results were reported as mean differences with 95% CIs. Given the ordinal nature of Likert-scale data, Wilcoxon signed-rank tests were conducted as a sensitivity analysis. To account for the multiplicity of pairwise comparisons across the top 3 performing models, the Benjamini-Hochberg procedure was applied to control the false discovery rate and mitigate type I error. Consequently, statistical significance for all intermodel comparisons was defined as a false discovery rate–adjusted *P* value (q value)<.05. Subgroup analyses of final diagnosis were conducted according to cystoscopic finding categories and anatomic sites, following the same statistical procedures.

For interpretability, the mean Likert-scale score for each item was further converted into a binary satisfaction outcome: satisfactory if the mean score was >3 and unsatisfactory if ≤3. The satisfaction rate (percentage of satisfactory responses) was reported and used as a binary outcome in subsequent analyses.

The performance metrics for the classification tasks—including binary domains (lesion detection: present vs absent, biopsy indication: yes or no, and malignancy: yes or no) and the 7-class lesion classification—were derived from confusion matrices. Reported metrics included accuracy, sensitivity, specificity, positive predictive value, negative predictive value, Youden J, and the Matthews correlation coefficient (MCC) [23]. Youden J represents the overall diagnostic effectiveness of a test, defined as (sensitivity + specificity – 1), and reflects the balance between true-positive and true-negative rates. The MCC quantifies the overall agreement between predicted and actual classifications by incorporating all 4 components of the confusion matrix (true or false positives and negatives). Metric comparisons were conducted using the chi-square test.

For the 7-class task (n=113), models were instructed to select exactly 1 forced-choice label from the prespecified options. Outputs failing to provide a single permissible choice (eg, refusals such as “I could not answer this question”) were coded as invalid. To ensure a consistent head-to-head comparison and minimize selection bias, the primary (strict) analysis used an intent-to-treat approach: invalid outputs were retained in the denominator and treated as incorrect predictions. However, because an invalid output does not necessarily reflect an incorrect diagnosis and may instead represent abstention—potentially safer than guessing in a human-in-the-loop workflow—we conducted a secondary sensitivity analysis, recalculating performance metrics conditional on valid responses only. All statistical analyses were conducted using SAS software (version 9.4; SAS Institute Inc).

### Ethical Considerations

The Research Ethics Committee A of National Taiwan University Hospital determined that this study was exempt from human participant research (NTUH-REC 202507210W). Informed consent was waived because this study involved a secondary analysis of deidentified cystoscopic images with no patient contact or intervention; for publicly available or published images, consent for the original collection followed the source publication, and the exemption permitted secondary analysis without additional consent. All images were deidentified, stored on access-controlled institutional systems, and reported only in aggregate. No participants were recruited, and no compensation was provided; all figures were reviewed to ensure no individual is identifiable.

## Results

### Distribution of the Whole Test Set and the Tumor-like Lesion Subset

Among 401 cystoscopic images, most originated from the bladder (n=329), followed by the prostate (n=41) and urethra (n=31). Abnormal findings were present in 322 (80.3%) images. The most common categories were tumor or neoplasm (n=126), structural or outlet abnormalities (n=76), inflammatory or reactive changes (n=69), deposits or foreign bodies (n=43), and vascular lesions (n=8); 79 (19.7%) images showed normal anatomy (Table 1). Table 1 provides the detailed distribution of finding subcategories, reflecting diagnostic diversity and difficulty.

**Table 1:** Detailed distribution of cystoscopic finding subcategories in the whole test dataset (N=401). This table provides a comprehensive breakdown of all observed cystoscopic findings across 3 hierarchical levels (normality, categories, and subcategories) and anatomic sites (bladder, prostate, and urethra). Values are presented as n (intracategory %); percentages represent the proportion of each subcategory within its respective parent category. The inclusion of both benign and malignant findings illustrates the heterogeneity of endoscopic presentations and underscores the diagnostic complexity represented in the dataset.

Anatomic site				Bladder		Prostate		Urethra		Total
Finding normality, categories, and subcategories, n (intracategory %)										
Abnormal				263		35		24		322
	Tumor or neoplasm			114 (100)		—		12 (100)		126
		Bladder polyp	7 (6.1)		—		—		7	
		Suspected bladder CIS^a^	17 (14.9)		—		—		17	
		Suspected nephrogenic adenoma	2 (1.8)		—		—		2	
		Papilloma	12 (10.5)		—		—		12	
		Papillary urothelial carcinoma	52 (45.6)		—		—		52	
		Nonurothelial carcinoma	18 (15.8)		—		—		18	
		Endometriosis	5 (4.4)		—		—		5	
		Teratoma	1 (0.9)		—		—		1	
		Urethral polyp	—		—		4 (33.3)		4	
		Urethral tumor	—		—		8 (66.7)		8	
	Inflammation or reaction			67 (100)		1 (100)		1 (100)		69
		Bladder amyloidosis	4 (6.0)		—		—		4	
		Bladder keratinizing	6 (9.0)		—		—		6	
		Bladder malakoplakia squamous metaplasia	5 (7.5)		—		—		5	
		Bladder mucosal break	1 (1.5)		—		—		1	
		Cystitis	26 (38.8)		—		—		26	
		Hemorrhagic cystitis	5 (7.5)		—		—		5	
		Suspected IC^b^	10 (14.9)		—		—		10	
		Suspected radiation cystitis	7 (10.4)		—		—		7	
		Suspected Schistosomiasis	3 (4.5)		—		—		3	
		Urethritis	—		1 (100)		1 (100)		2	
	Deposits or foreign bodies			39 (100)		2 (100)		2 (100)		43
		Bladder encrustation	5 (12.8)		—		—		5	
		Blood clot	12 (30.8)		1 (50)		1 (50)		14	
		Foreign body	7 (17.9)		—		1 (50)		8	
		Stone	15 (38.5)		1 (50)		—		16	
	Structure or outlet			35 (100)		32 (100)		9 (100)		76
		Bladder diverticulum	4 (11.4)		—		—		4	
		Bladder neck contracture	4 (11.4)		—		—		4	
		Bladder scar	12 (34.3)		—		—		12	
		Bladder trabeculation	2 (5.7)		—		—		2	
		Vesicoureteral reflux	4 (11.4)		—		—		4	
		Ureterocele	6 (17.1)		—		—		6	
		Suspected fistula	3 (8.6)		—		2 (22.2)		5	
		Prostate enlargement	—		31 (96.9)		—		31	
		Prostatic cyst	—		1 (3.1)		—		1	
		Urethra stricture	—		—		4 (44.4)		4	
		Urethral cyst	—		—		1 (11.1)		1	
		Urethral trauma	—		—		2 (22.2)		2	
	Vascularity			8 (100)		—		—		8
		Bladder hemangioma	1 (12.5)		—		—		1	
		Bladder telangiectasia	4 (50.0)		—		—		4	
		Bladder varices	3 (37.5)		—		—		3	
Normal				66		6		7		79

^a^CIS: carcinoma in situ.

^b^IC: interstitial cystitis.

The tumor-like lesion subset included 113 visually and pathologically similar images spanning both benign and malignant lesions: cystitis (n=18), polyps (n=7), papilloma (n=12), pUC (n=20), CIS (n=17), non-U Ca (n=17), and NOTA (n=22) (Table 2).

**Table 2:** Distribution of the tumor-like lesion subset (n=113) used for the 7-class lesion classification task, representing a focused subset of the whole test dataset. The tumor-like lesion subset comprised 18 cystitis (15.9%), 7 polyps (6.2%), 12 papilloma (10.6%), 20 papillary urothelial carcinoma (pUC; 17.7%), 17 carcinoma in situ (CIS; 15%), 17 non-urothelial carcinoma (non-U Ca; 15%), and 22 none of the above (NOTA; 19.5%).

Lesion type	Value, n (%)
Cystitis	18 (15.9)
Polyps	7 (6.2)
Papilloma	12 (10.6)
pUC^a^	20 (17.7)
CIS^b^	17 (15)
Non-U Ca^c^	17 (15)
NOTA^d^	22 (19.5)
Total	113 (100)

^a^pUC: papillary urothelial carcinoma.

^b^CIS: carcinoma in situ.

^c^Non-U Ca: non-urothelial carcinoma.

^d^NOTA: none of the above.

### Comparative Mean Scores of LLMs in Image Interpretation

**Figure 2:**
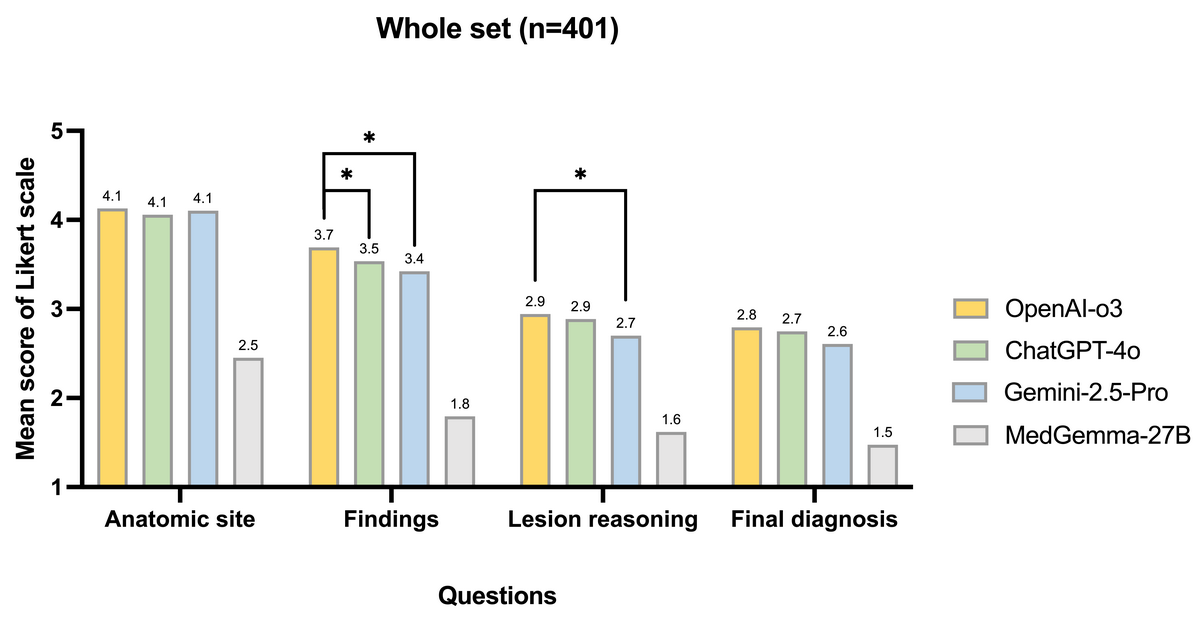
Comparative mean scores of multimodal large language models (MM-LLMs) for cystoscopic image interpretation across 4 question domains in the whole test set. Asterisks denote statistically significant pairwise differences among the top 3 models. *q<0.05, where q is the false discovery rate (FDR)-adjusted *P* value.

Among the whole test set (n=401), MM-LLMs demonstrated progressively lower performance as task complexity increased (Table 3 and Figure 2). Mean Likert-scale scores declined from anatomic site recognition (≈ 4.1) to findings (≈ 3.4-3.7), lesion reasoning (≈ 2.7-2.9), and final diagnosis (≈ 2.6-2.8). OpenAI-o3, ChatGPT-4o, and Gemini 2.5 Pro achieved comparable accuracy in anatomical localization, while OpenAI-o3 showed the highest overall consistency and clarity in lesion description. Statistically significant differences emerged in the findings, lesion reasoning, and final diagnosis domains, in which OpenAI-o3 outperformed Gemini 2.5 Pro and the medical-specific MedGemma-27B. Notably, MedGemma-27B lagged substantially behind the general-purpose MM-LLMs across all categories, suggesting that its limited training scope constrained both descriptive precision and diagnostic reasoning. These results indicate that reasoning-optimized general-purpose MM-LLMs currently outperform open-source, domain-specific models in free-text cystoscopic interpretation tasks.

**Table 3:** Performance of 4 multimodal large language models (MM-LLMs) in cystoscopic image interpretation, presented as mean Likert scores and SDs across open-ended questions and final-diagnosis subgroups.

Question and subgroup				Value		OpenAI-o3, mean (SD)		ChatGPT-4o, mean (SD)		Gemini 2.5 Pro, mean (SD)		MedGemma-27B, mean (SD)
**Whole test set (n=401)**												
	Questions											
		Q1: anatomic site	401		4.13 (1.24)		4.06 (1.20)		4.10 (1.21)		2.45 (1.37)	
		Q2: findings	401		3.69 (1.23)		3.54 (1.25)		3.42 (1.30)		1.80 (1.03)	
		Q4: lesion reasoning	401		2.94 (1.57)		2.89 (1.49)		2.70 (1.48)		1.62 (1.11)	
		Q5: final diagnosis	401		2.79 (1.59)		2.75 (1.51)		2.61 (1.51)		1.48 (0.93)	
**Q5. final diagnosis**												
	Subgrouping by findings											
		Tumor or neoplasm	126		3.12 (1.44)		3.32 (1.36)		3.07 (1.51)		2.06 (1.18)	
		Inflammation or reaction	69		2.34 (1.21)		2.82 (1.30)		2.87 (1.29)		1.19 (0.45)	
		Deposits or foreign bodies	43		2.77 (1.59)		2.97 (1.52)		2.64 (1.64)		1.07 (0.26)	
		Structure or outlet	76		1.71 (1.13)		1.45 (0.69)		2.45 (1.67)		1.03 (0.10)	
		Vascularity	8		2.42 (1.05)		2.75 (0.71)		2.75 (0.87)		1.29 (0.70)	
		Normal	79		3.79 (1.76)		2.92 (1.81)		1.76 (1.13)		1.49 (1.04)	
	Subgrouping by anatomic site											
		Bladder	329		3.04 (1.56)		3.06 (1.46)		2.64 (1.47)		1.56 (1.00)	
		Prostate	41		1.45 (1.02)		1.24 (0.62)		2.95 (1.79)		1.09 (0.31)	
		Urethra	31		2 (1.34)		1.48 (0.91)		1.80 (1.27)		1.12 (0.38)	

Mean-score pairwise comparisons among models (mean-score deltas) are provided in Table S2 in Multimedia Appendix 1. A 0.2-point difference on the 5-point Likert score corresponds approximately to a 5-point difference on a 100-point scale. The matrix of column-row differences confirmed OpenAI-o3’s edge across open-question domains. Versus Gemini 2.5 Pro, OpenAI-o3 scored higher by +0.27 on findings (q=0.002), +0.24 on lesion reasoning (q=0.047), +0.03 on anatomic site, and +0.19 on final diagnosis (not significant). Against ChatGPT-4o, OpenAI-o3 held a small but consistent advantage on findings (+0.15, q=0.004) with near-parity on anatomic site, lesion reasoning, and final diagnosis (+0.07, +0.06, +0.04; not significant). All general-purpose models substantially outperformed the medical-specific MedGemma-27B; OpenAI-o3’s margins were +1.68 (anatomic site), +1.90 (findings), +1.32 (lesion reasoning), and +1.32 (final diagnosis), all q<0.001. Taken together, these deltas indicate that OpenAI-o3 is the most reliable free-text interpreter, with the largest, statistically robust gains in content-heavy domains (Findings → Reasoning → Diagnosis). The significance pattern of the Wilcoxon signed-rank tests was consistent with that of the paired *t* tests.

Intraclass correlation coefficients demonstrated excellent interrater reliability across both model and question domains. Of the 16 intraclass correlation coefficient values, 14 ranged from 0.82 to 0.94, indicating high consistency among raters (Table S3 in Multimedia Appendix 1).

### Model Satisfaction Rates

**Figure 3:**
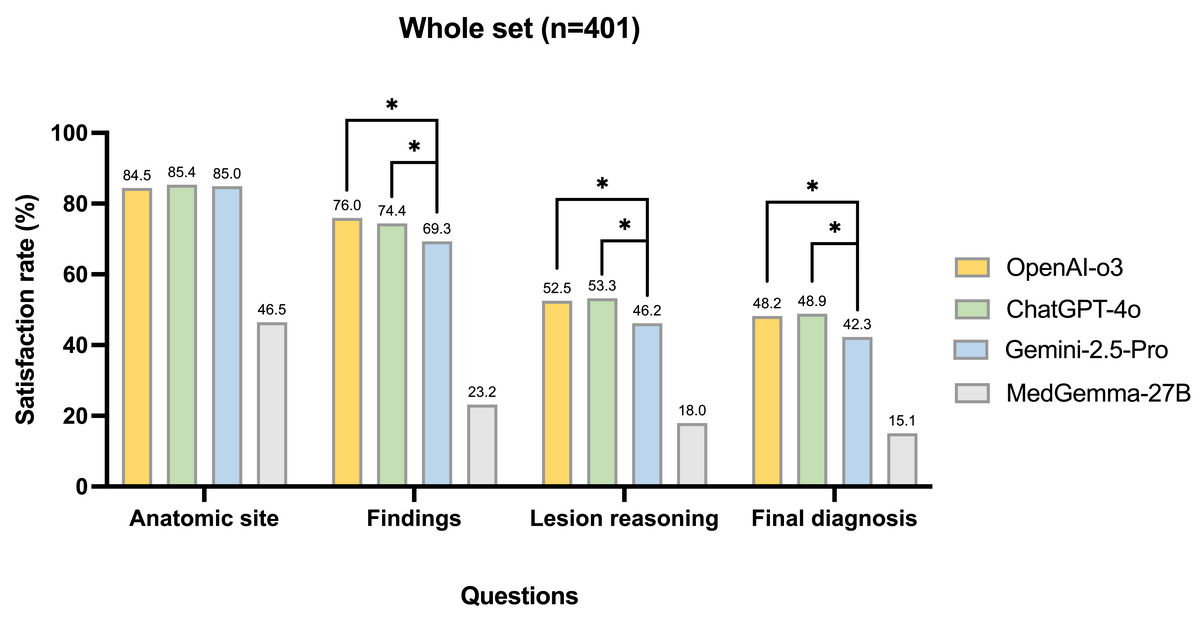
Comparative satisfaction rates (% of cases with mean score >3) of multimodal large language models (MM-LLMs) for cystoscopic image interpretation across 4 question domains. Asterisks denote significance for pairwise comparisons between the top 3 models. *q<0.05, where q is the false discovery rate (FDR)-adjusted *P* value.

Satisfaction rates for each question across models closely paralleled mean scores of the Likert scale (Figure 3). Overall, satisfaction ranked anatomic site > findings > lesion reasoning ≈ final diagnosis, consistent with mean-score trends. Anatomic site showed uniformly high satisfaction for the top 3 models (339/401, ≈85%), while MedGemma-27B was much lower (184/401, 46%). For findings, OpenAI-o3 (305/401, 76%) and ChatGPT-4o (297/401, 74%) outperformed Gemini 2.5 Pro (277/401, 69%) and MedGemma-27B (92/401, 23%; all q<0.01). In lesion reasoning, OpenAI-o3 (211/401, 53%) and ChatGPT-4o (201/401, 53%) outperformed Gemini 2.5 Pro (184/401, 46%; q=0.003 and q=0.002 vs Gemini 2.5 Pro, respectively), while MedGemma-27B again had the lowest performance (72/401, 18%). For final diagnosis, satisfaction was lowest overall but remained higher for OpenAI-o3 and ChatGPT-4o (192/401, ≈48%) than for Gemini 2.5 Pro (168/401, 42%) or MedGemma-27B (60/401, 15%).

### Subgroup Analysis of Final Diagnosis

**Figure 4:**
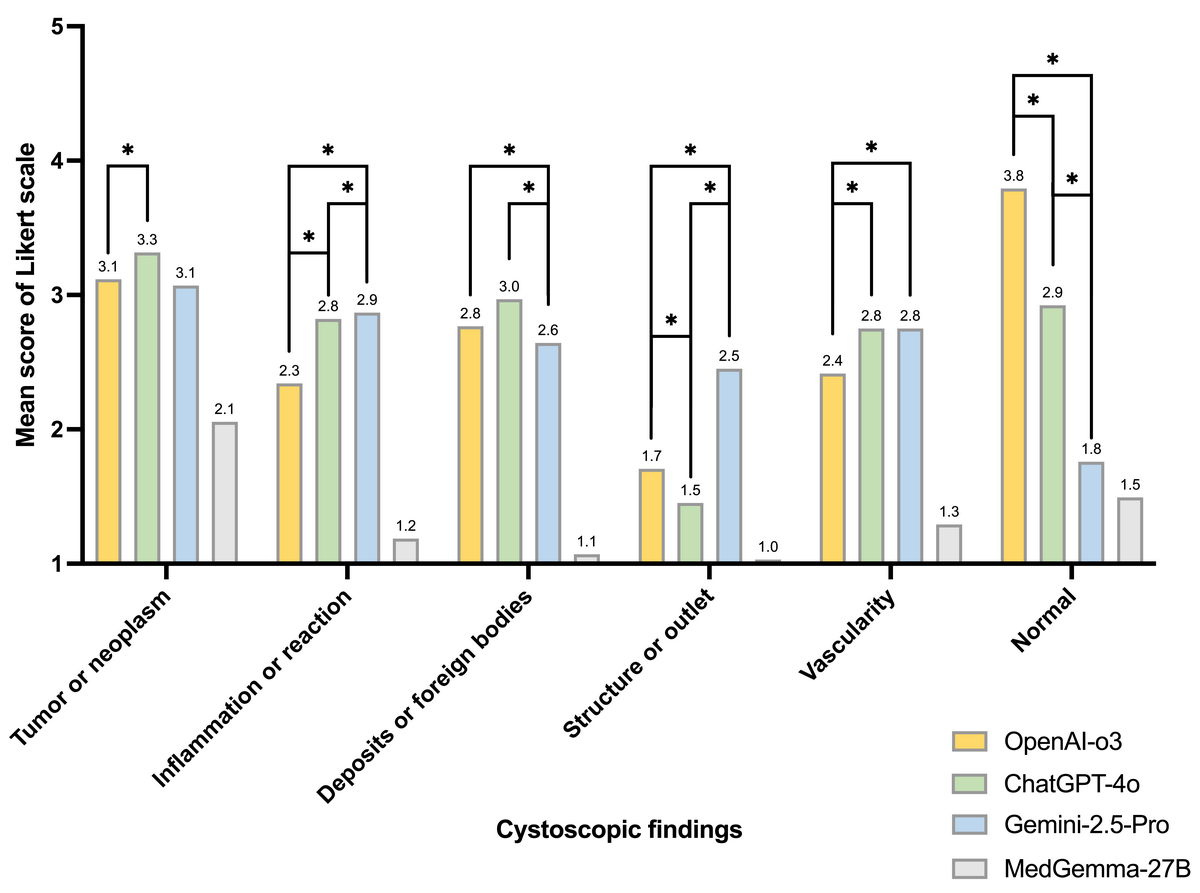
Subgroup analysis of mean final diagnosis scores across 6 cystoscopic finding categories (tumor or neoplasm, inflammation, deposits, structure, vascularity, and normal) for 4 multimodal large language models (MM-LLMs). Asterisks denote significance for pairwise comparisons between the top 3 models. *q<0.05, where q is the false discovery rate (FDR)-adjusted *P* value.

When mean scores for final diagnosis were stratified by finding category (Figure 4 and Table 3), the largest intermodel differences occurred in the normal and structure or outlet groups. OpenAI-o3 achieved the highest score for normal findings (3.79), significantly outperforming ChatGPT-4o (2.92) and Gemini 2.5 Pro (1.76; q<0.001). Conversely, Gemini 2.5 Pro scored best for structure or outlet findings (2.45), exceeding OpenAI-o3 (1.71) and ChatGPT-4o (1.45; q<0.001). For tumor or neoplasm, ChatGPT-4o slightly surpassed OpenAI-o3 (3.32 vs 3.12; q=0.02), with Gemini 2.5 Pro showing comparable performance (3.07). Inflammation or reaction and vascularity categories both favored ChatGPT-4o and Gemini 2.5 Pro over OpenAI-o3, whereas deposits or foreign bodies showed minimal differences among models.

**Figure 5:**
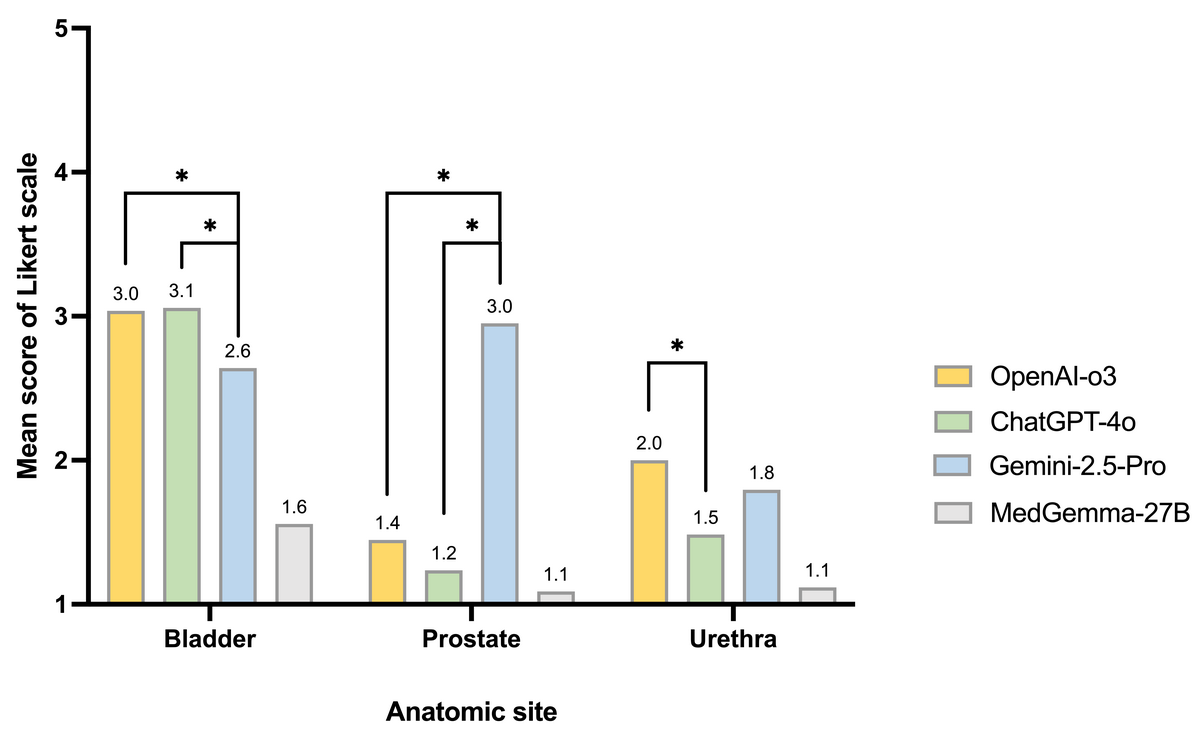
Subgroup analysis of mean final diagnosis scores across 3 anatomic sites (bladder, prostate, and urethra) for 4 multimodal large language models (MM-LLMs). Asterisks denote significance for pairwise comparisons between the top 3 models. *q<0.05, where q is the false discovery rate (FDR)-adjusted *P* value.

Performance also varied by anatomic site (Figure 5 and Table 3). Gemini 2.5 Pro performed best in the prostate (2.95), significantly exceeding OpenAI-o3 (1.45) and ChatGPT-4o (1.24; q<0.001). In the bladder, OpenAI-o3 (3.04) and ChatGPT-4o (3.06) outperformed Gemini 2.5 Pro (2.64; q<0.001). For the urethra, OpenAI-o3 (2) exceeded ChatGPT-4o (1.48; q=0.02), with Gemini 2.5 Pro intermediate (1.80). These site-specific trends suggest complementary strengths: Gemini 2.5 Pro performs relatively better in structure-dominated prostate views, whereas OpenAI-o3 and ChatGPT-4o perform best in bladder-focused interpretation.

### Clinically Relevant Binary Endpoints

For the lesion-detection task in the whole test set, OpenAI-o3 achieved the highest overall performance, with an accuracy of 88.3%, a Youden J of 0.650, and an MCC of 0.635, followed by ChatGPT-4o and Gemini 2.5 Pro, while MedGemma-27B performed the lowest (Table 4). OpenAI-o3 demonstrated the most balanced profile (sensitivity 92% and specificity 73.1%), whereas ChatGPT-4o showed higher sensitivity but lower specificity (94.4% vs 44.2%). Gemini 2.5 Pro exhibited an extreme trade-off—maximal sensitivity (99.7%) but very low specificity (10.3%). MedGemma-27B produced the weakest results overall (accuracy 45.6%, Youden J –0.103, and MCC –0.081).

**Table 4:** Binary classification performance of 4 multimodal large language models (MM-LLMs) in clinically relevant cystoscopic endpoints (strict analysis).

Task and MM-LLM^a^					VRR^b^ (%)			Acc^c^ (%)			Sen^d^ (%)			Spec^e^ (%)			PPV^f^ (%)			NPV^g^ (%)			Youden J^h^			MCC^i^
**Whole test set (n=401)**																										
	Lesion detection (present vs absent)																									
		OpenAI-o3	100			88.3			92.0			73.1			93.4			68.7			0.650			0.635		
		ChatGPT-4o	100			84.7			94.4			44.2			87.6			65.4			0.386			0.452		
		Gemini 2.5 Pro	100			82.3			99.7			10.3			82.1			88.9			0.100			0.266		
		MedGemma-27B	100			45.6			46.1			43.6			77.2			16.4			-0.103			-0.081		
**Tumor-like lesion subset (n=113)**																										
	Biopsy indication (yes or no): zero-shot prompting																									
		OpenAI-o3	99.1			73.5			82.2			57.5			77.9			63.9			0.397			0.407		
		ChatGPT-4o	92.9			69.0			91.8			27.5			69.8			64.7			0.193			0.258		
		Gemini 2.5 Pro	100			70.8			86.3			42.5			73.3			63.0			0.288			0.323		
		MedGemma-27B	100			65.5			97.3			7.5			65.7			60.0			0.048			0.111		
	Biopsy indication (yes or no): in-context learning																									
		OpenAI-o3	100			76.1			80.8			67.5			81.9			65.6			0.483			0.481		
		ChatGPT-4o	98.2			69.0			89.0			32.5			70.7			61.9			0.215			0.265		
		Gemini 2.5 Pro	100			69.0			75.3			57.5			76.4			56.1			0.328			0.327		
		MedGemma-27B	100			60.2			93.2			0.0			63.0			0.0			-0.069			-0.159		
	Presence of malignancy (yes or no): zero-shot prompting																									
		OpenAI-o3	99.1			62.8			79.6			47.5			58.1			71.8			0.271			0.285		
		ChatGPT-4o	92.9			55.8			81.5			32.2			52.4			65.5			0.137			0.157		
		Gemini 2.5 Pro	100			61.1			87.0			37.3			56.0			75.9			0.243			0.278		
		MedGemma-27B	100			57.5			72.2			44.1			54.2			63.4			0.163			0.169		
	Presence of malignancy (yes or no): in-context learning																									
		OpenAI-o3	100			63.7			70.4			57.6			60.3			68.0			0.280			0.282		
		ChatGPT-4o	98.2			59.3			61.1			57.6			56.9			61.8			0.187			0.187		
		Gemini 2.5 Pro	100			62.0			66.7			57.6			59.0			65.4			0.243			0.244		
		MedGemma-27B	100			52.2			87.0			20.3			50.0			63.2			0.074			0.099		

^a^MM-LLM: multimodal large language model.

^b^VRR: valid response rate. Valid response rate = (total - invalid) / total. Invalid denotes outputs failing to provide a single permissible choice.

^c^Acc: accuracy.

^d^Sen: sensitivity.

^e^Spe: specificity.

^f^PPV: positive predictive value.

^g^NPV: negative predictive value.

^h^Youden J: Youden J Index.

^i^MCC: Matthews correlation coefficient.

In the tumor-like lesion subset (n=113), OpenAI-o3 again achieved the highest Youden J and MCC performance for both biopsy-indication and malignancy endpoints, followed by Gemini 2.5 Pro and ChatGPT-4o, with MedGemma-27B lowest. For biopsy indication, OpenAI-o3 reached 73.5% accuracy (Youden J=0.397 and MCC=0.407), demonstrating the best specificity-sensitivity balance. ICL modestly improved specificity and accuracy (Table 4). For malignancy detection, OpenAI-o3 similarly performed best (accuracy 62.8%), followed by Gemini 2.5 Pro and ChatGPT-4o, whereas MedGemma-27B underperformed (Tables S4-S5 in Multimedia Appendix 1).

**Figure 6:**
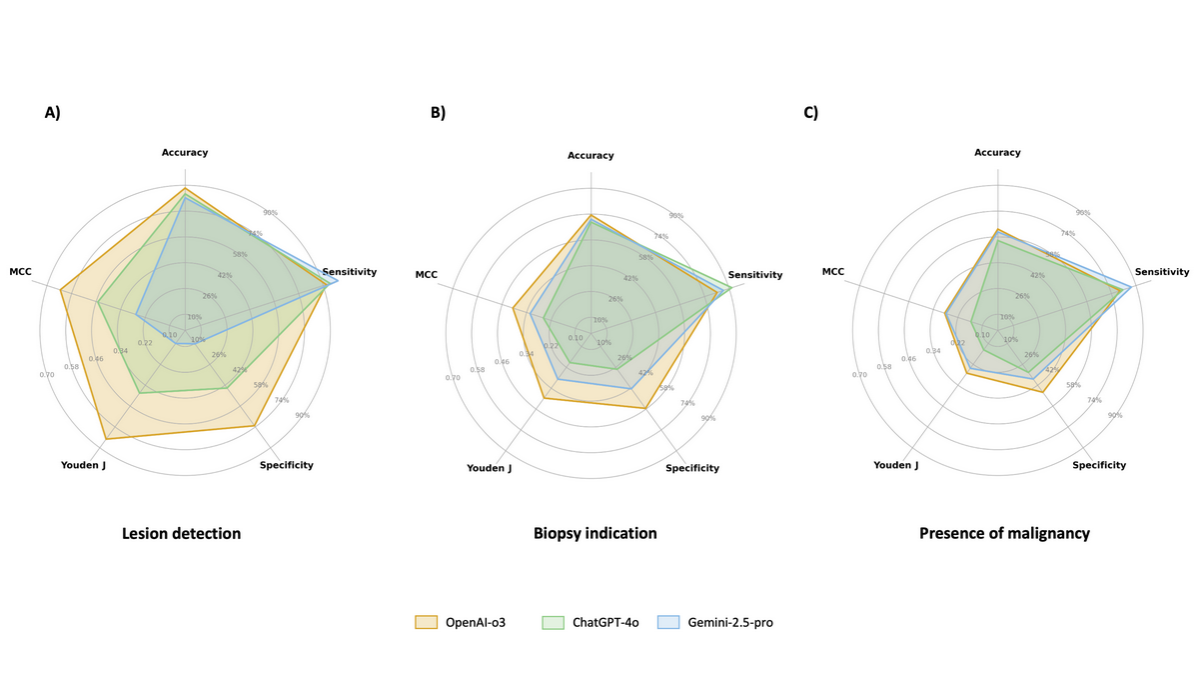
Radar charts illustrating diagnostic performance across 3 cystoscopic endpoints in classification tasks. Panels represent (A) lesion detection (presence vs absence), (B) biopsy indication (yes vs no), and (C) presence of malignancy (yes vs no). Five key metrics are visualized: accuracy, sensitivity, specificity, Youden J index, and Matthews correlation coefficient (MCC).

Overall, OpenAI-o3 demonstrated the most balanced diagnostic performance across all 3 binary endpoints, consistently achieving the highest Youden J, largely attributable to its superior specificity (Figure 6). For lesion detection, specificity reached 73.1%, significantly outperforming ChatGPT-4o (44.2%; q<0.001) and Gemini 2.5 Pro (10.3%; q<0.001; Figure 6A). In biopsy indication, specificity was 57.5%, again higher than ChatGPT-4o (27.5%; q<0.001) and Gemini 2.5 Pro (42.5%; q=0.047; Figure 6B). In malignancy prediction, OpenAI-o3 maintained the highest specificity (47.5%) compared to ChatGPT-4o (32.2%; q=0.042) and Gemini 2.5 Pro (37.3%; q=0.21; Figure 6C). In contrast, MedGemma-27B demonstrated limited generalizability despite its medical-domain optimization.

### Seven-Class Tumor-Like Lesion Classification

For the 7-class classification (n=113), OpenAI-o3 remained the top-performing model, although overall accuracy was modest, improving only slightly with ICL (microaverage accuracy from 40.7% to 46% and MCC from 0.311 to 0.370) (Table 5). Class-level performance was heterogeneous. In the zero-shot setting, malignant categories (pUC and CIS) achieved relatively balanced sensitivity and specificity, whereas benign lesions (cystitis, polyps, and papilloma) showed high specificity but low sensitivity. Notably, non-U Ca were not recognized. ICL mainly adjusted the sensitivity-specificity balance—enhancing detection of polyps, papilloma, and NOTA while slightly reducing sensitivity for cystitis and CIS. However, non-U Ca remained unrecognized.

**Table 5:** Confusion matrix outlining the performance of OpenAI-o3 in 7-class tumor-like lesion classification under zero-shot and in-context learning prompting (strict analysis).

			Actual																				Total		
			Cystitis		Polyps			Papilloma		pUC^a^				CIS^b^			Non-U Ca^c^			NOTA^d^					
**Predicted**																									
	Zero-shot prompting																								
		Cystitis	6		1			1		0				2			1			3			14		
		Polyps	1		1			0		0				0			1			0			3		
		Papilloma	0		0			0		0				0			0			0			0		
		pUC	2		4			11		20				0			14			7			58		
		CIS	5		0			0		0				9			0			2			16		
		Non-U Ca	0		0			0		0				0			0			0			0		
		NOTA	4		1			0		0				5			1			10			21		
		Invalid^e^	0		0			0		0				1			0			0			1		
		Total	18		7			12		20				17			17			22			113		
	ICL^f^ prompting																								
		Cystitis	6		0			1		1				6			0			4			18		
		Polyps	0		3			0		0				0			3			0			6		
		Papilloma	0		0			3		0				0			0			0			3		
		pUC	2		4			8		19				0			12			4			49		
		CIS	7		0			0		0				7			0			0			14		
		Non-U Ca	0		0			0		0				0			0			0			0		
		NOTA	3		0			0		0				4			2			14			23		
		Total	18		7			12		20				17			17			22			113		
**Classification metrics**																									
	Zero-shot prompting																								
		Accuracy	82.3		92.9			89.4		66.4				86.7			85.0			79.6			40.7		
		Sensitivity	33.3		14.3			0		100				52.9			0			45.5			40.7		
		Specificity	91.6		98.1			100		59.1				92.7			100			87.9			90.3		
		PPV^h^	42.9		33.3			0		34.5				56.3			0			47.6			41.1		
		NPV^i^	87.9		94.5			89.4		100				91.8			85.0			87.0			90.1		
		Youden J^j^	0.249		0.124			0		0.591				0.456			0			0.334			0.310		
		MCC^k^	0.277		0.186			0		0.452				0.468			0			0.340			0.311		
	ICL prompting																								
		Accuracy	78.8		93.8			92.0		72.6				85.0			85.0			85.0			46.0		
		Sensitivity	33.3		42.9			25.0		95.0				41.2			0			63.6			46.0		
		Specificity	87.4		97.2			100		67.7				92.7			100			90.1			91.0		
		PPV	33.3		50.0			100		38.8				50			0.0			60.9			46.0		
		NPV	87.4		96.3			91.8		98.4				89.9			85.0			91.1			91.0		
		Youden J	0.207		0.400			0.250		0.627				0.339			0			0.538			0.370		
		MCC	0.207		0.430			0.479		0.483				0.368			0			0.529			0.370		

^a^pUC: papillary urothelial carcinoma.

^b^CIS: carcinoma in situ.

^c^Non-U Ca: non-urothelial carcinoma.

^d^NOTA: none of the above.

^e^Invalid: model outputs failing to provide a single permissible choice.

^f^ICL: in-context learning.

^g^AVG: microaverage.

^h^PPV: positive predictive value.

^i^NPV: negative predictive value.

^j^Youden J: Youden J Index.

^k^MCC: Matthews correlation coefficient.

The other 3 models performed suboptimally and showed limited responsiveness to ICL (Tables S6-S8 in Multimedia Appendix 1). In the zero-shot setting, microaveraged accuracy ranked as follows: 36.3% (Gemini 2.5 Pro); 31.9% (ChatGPT-4o), 28.3% (MedGemma-27B), with Youden J and MCC both 0.164-0.257. Under ICL, accuracy was similar or slightly lower—34.5% (Gemini 2.5 Pro), 31% (ChatGPT-4o), and 27.4% (MedGemma-27B)—with minimal shifts in Youden J and MCC (0.153-0.236). Class-wise patterns were consistent: malignant categories (pUC, CIS, and non-U Ca) showed the most balanced sensitivity-specificity trade-offs, whereas benign entities (cystitis, polyps, and papilloma) had low sensitivity but high specificity.

### Analysis of the NOTA Category

The NOTA category represents a unique “negative exclusion” challenge. Previous research indicates that LLMs often exhibit a bias toward positive selection, struggling to confidently select “None of the Above” even when accurate [24,25]. Our results show that this bias is pervasive, affecting models across different architectures (Tables S6-S8). Despite being a reasoning-optimized model, Gemini 2.5 Pro aligned with the general-purpose ChatGPT-4o and the medical-specific MedGemma-27B in exhibiting a “high specificity, low sensitivity” pattern. Specifically, Gemini 2.5 Pro and ChatGPT-4o achieved high specificity (>97%) but low sensitivity (9.5%-27.3%) across prompting strategies. The open-weight MedGemma-27B exhibited the most severe manifestation of this bias: while its specificity remained high (98.9%), its sensitivity was only 13.6% in the zero-shot setting and collapsed to 0% under ICL prompting. This indicates that for models unable to effectively leverage negative logic, added textual context may inadvertently reinforce positive selection bias.

A distinct divergence was observed between the 2 reasoning-optimized models. In contrast to Gemini 2.5 Pro, OpenAI-o3 demonstrated superior handling of exclusion (Table 5). It achieved a significantly higher baseline sensitivity of 45.5% in the zero-shot setting. Moreover, while ICL yielded negligible or detrimental effects for the other 3 models, OpenAI-o3’s sensitivity surged to 63.6% under ICL prompting. This suggests that OpenAI-o3’s specific implementation of chain-of-thought reasoning is critical for overcoming the standard positive selection bias, allowing for robust diagnosis through exclusion where other reasoning and general models failed.

### Sensitivity Analysis: Conditional on Valid Responses

In the tumor-like lesion classification task, invalid (refusal) outputs were uncommon: valid-response rates were ≈100% for OpenAI-o3, Gemini 2.5 Pro, and MedGemma-27B, whereas ChatGPT-4o had the highest invalid rate (7.1%) in zero-shot prompting. While excluding invalid responses can inflate performance (introducing optimistic bias) relative to the strict analysis, invalid outputs can be interpreted clinically as abstention, which may be safer than guessing in a human-in-the-loop workflow because it prompts clinician confirmation. Accordingly, we report conditional-on-valid performance to better reflect accuracy when the model provides a valid output (Table S9 in Multimedia Appendix 1).

ChatGPT-4o showed the largest strict vs conditional-on-valid differences, consistent with its lower valid-response rate (Table 4 vs Table S9 in Multimedia Appendix 1). Accuracy and Youden J increased from 69% and 0.193 to 74.3% and 0.267 for biopsy indication and from 55.8% and 0.137 to 60% and 0.205 for malignancy. Seven-class changes were small, most notably higher sensitivity for NOTA (from 13.6% to 15%) and cystitis (from 11.1% to 14.3%), with microaverage sensitivity rising from 31.9% to 34.3%.

ICL-focused takeaway across models was that text-only ICL chiefly reweighted sensitivity-specificity rather than boosting overall accuracy; modest gains in benign or NOTA recognition were offset by reduced sensitivity in key malignant classes.

## Discussion

### Principal Findings

This study is the first to benchmark SOTA MM-LLMs for cystoscopic interpretation under a clinician-defined stress test with rare and diagnostically difficult lesions. The rigorous, blinded design enabled objective assessment of interpretive reasoning and classification. Outputs were also mapped to actionable binary endpoints and used to quantify the incremental effect of text-based ICL over zero-shot prompting, thereby revealing both strengths and current limitations of MM-LLMs in real-world clinical tasks.

Overall, OpenAI-o3 demonstrated superior performance, followed by ChatGPT-4o and Gemini 2.5 Pro, with MedGemma-27B showing the most limited capabilities. The results revealed a progressive decline in model performance as diagnostic complexity increased—from anatomical recognition to higher-order diagnostic synthesis. While models showed meaningful strength in visual recognition and descriptive reporting, performance in challenging differential diagnosis remained modest, suggesting that current MM-LLMs function best as assistive rather than autonomous diagnostic tools at present.

### Image Interpretation and Lesion Classification Tasks

The free-text interpretation task assessed 4 domains of increasing complexity—anatomic site, findings, lesion reasoning, and final diagnosis—simulating real-world diagnostic synthesis that integrates visual recognition with clinical reasoning. Task satisfaction declined with increasing complexity, from anatomic localization (~85%) to definitive diagnosis (~45%). OpenAI-o3 and ChatGPT-4o consistently outperformed Gemini 2.5 Pro and MedGemma-27B, though with distinct profiles: OpenAI-o3 produced concise, accurate descriptions with coherent diagnostic impressions and high specificity for normal anatomy, while ChatGPT-4o showed greater sensitivity for inflammatory and vascular findings. In contrast, Gemini 2.5 Pro often overcalled minor irregularities but performed better on prostate lesions, likely reflecting prostate-predominant pretraining. These discrepancies indicate that MM-LLM behavior depends not only on recognition accuracy but also on underlying reasoning logic, diagnostic thresholds, and domain-specific pretraining.

Regarding clinical decision endpoints, the goal of cystoscopy is to identify abnormal lesions—particularly malignancies—so that biopsies are performed when necessary while avoiding unnecessary procedures that increase cost and risk. Thus, lesion detection, biopsy indication, and malignancy presence were defined as key clinical endpoints. OpenAI-o3 achieved the most balanced performance across sensitivity, specificity, and Youden J, outperforming ChatGPT-4o and Gemini 2.5 Pro, especially in specificity, by accurately distinguishing normal from malignant cases—supporting appropriate biopsy decision-making. These findings highlight the importance of calibrating operating points to clinical priorities and suggest that MM-LLMs, particularly OpenAI-o3, can aid cystoscopic decision-making when optimized for an appropriate specificity-sensitivity balance.

The 7-class lesion classification task evaluated each model’s ability to distinguish visually and pathologically similar tumor-like lesions. Models performed best on prevalent malignant lesions (pUC) but struggled with benign mimickers (polyps and papilloma) and rare entities (non-U Ca), often misclassified as pUC—reflecting limited pretraining exposure to uncommon classes. As shown in Table 5**,** 14 of 17 non-U Ca cases (82.4%) were predicted as pUC, a much more prevalent bladder tumor. One plausible explanation is that LLMs exhibit a tendency to choose majority or high-frequency labels in multiple-choice settings. When pretraining class distributions are imbalanced, the token sequences corresponding to common options (eg, “pUC”) can carry higher previous probabilities, biasing the model toward these answers irrespective of correctness. This phenomenon is often described as majority-label bias or common-token bias [26].

On the other hand, the frequent misclassification of papilloma as pUC highlights the inherent challenge of distinguishing these entities based solely on cystoscopic appearance—a difficulty shared by human experts. Rather than indicating model failure, these confusion patterns reflect the substantial macroscopic overlap between papilloma and low-grade pUC. While visual distinction remains experimental, our study addressed this clinical reality by grouping both entities under the “Biopsy Indicated” category in the binary endpoint analysis. In that context, the models successfully flagged these lesions for histologic confirmation, aligning with standard safety protocols despite the specific classification ambiguity. CIS, a flat, high-grade, non-invasive UC subtype, was handled well by OpenAI-o3, achieving strong results (accuracy 86.7, sensitivity 52.9, specificity 92.7, and Youden J 0.46) despite diagnostic difficulty. Overall, OpenAI-o3 showed the most balanced performance, particularly excelling in benign and NOTA classifications, achieving higher specificity than ChatGPT-4o and Gemini 2.5 Pro.

### Performance Disparity and the Role of the Open-Weight Baseline

Although MedGemma-27B is a medical-specific model, its performance trailed behind the general-purpose proprietary models (OpenAI-o3, ChatGPT-4o, and Gemini 2.5 Pro). This gap can be attributed to 2 primary factors: domain-specific data misalignment and model scale. First, contrary to the expectation that a medical model should inherently outperform general models, MedGemma’s training distribution did not encompass the specific modality of cystoscopy. While its multimodal components (SigLIP encoder) were rigorously pretrained on diverse medical datasets—including chest X-rays, dermatology images, ophthalmology images, and histopathology slides—endoscopic imagery was notably absent from its pretraining corpus. Consequently, the model faced a “zero-shot” challenge in a domain it had not explicitly learned, whereas the massive general-purpose models likely benefited from broader exposure to endoscopic images present in their web-scale training data.

Second, as a local and open-weight baseline, MedGemma-27B (≈27B parameters) operates with significantly constrained capacity compared to the proprietary SOTA architectures. It lacks the extensive parameter count, training budget, and chain-of-thought optimization that allow models such as OpenAI-o3 to generalize across unseen tasks. Therefore, our intent is not to claim parity with these massive systems, but to establish a transparent performance floor for open-weight deployment. Despite the lower accuracy in this zero-shot setting, MedGemma-27B remains a critical benchmark for institutions requiring on-premise, privacy-preserving solutions. Its performance represents the current starting point for future adaptation, such as LoRA fine-tuning or retrieval-augmented prompting, rather than a direct competitor in raw reasoning capability.

### Comparing With Previous Studies

Guo et al. [17] reported comparable findings when evaluating ChatGPT-4V (OpenAI) and Claude-3.5 (Anthropic) on 603 cystoscopic images, achieving accuracies of 82.8% and 79.8% but with marked variability across conditions. Both models performed well for cystitis and bladder tumors but poorly for BPH and normal structures, indicating that general-purpose LLMs detect major lesions with high sensitivity but struggle with subtle findings. Similar variability has been observed in gastrointestinal endoscopy, where ChatGPT-4V showed mixed accuracy across lesion types and underperformed relative to tuned CNN models [27]. Recent work suggests that general multimodal models such as Gemini 2.5 Pro may even surpass specialized AI in certain “edge cases” [28]. Unlike task-specific systems, these models can simultaneously classify images and generate descriptive reasoning and management suggestions, offering value for clinical interpretation and education.

### Enhancing MM-LLM Performance in Medical-Specific Domain

Several in-domain strategies can improve general-purpose MM-LLMs without training from scratch, including ICL, contrastive pretraining, and retrieval augmentation.

In our study, text-only ICL with brief cystoscopic tumor descriptions had minimal impact, except for OpenAI-o3. Specificity rose slightly, and sensitivity improved for rare benign lesions, but this was offset by reduced sensitivity for malignant classes (pUC and CIS)—a trade-off in which stricter thresholds reduce false positives and unnecessary biopsies, but risk missed cancers. These findings indicate that text-only ICL confers minimal benefit for image-dominant tasks. Notably, OpenAI-o3 showed a modest gain in micro-average accuracy (0.41-0.46), driven mainly by NOTA, likely reflecting its reasoning-oriented architecture and more flexible use of ICL as contextual support.

A likely reason for the limited effect is insufficient visual grounding: text-only cues do not anchor the model’s attention to class-defining morphology. In fine-grained visual discrimination (eg, cystoscopic lesion typing), semantic hints (eg, “papillary fronds” and “flat erythematous base”) may not map reliably to visual features unless those associations were learned during pretraining; without image exemplars, the model’s visual reasoning remains underconstrained.

Beyond text-only prompts, few-shot image-text exemplars (multimodal ICL) can strengthen grounding and improve accuracy. Presenting paired lesion images with diagnoses exposes prototypical visual features and tightens the link between morphology and class semantics. Across histopathology imaging, image-text ICL has enabled ChatGPT-4V to approach or surpass task-specific classifiers with only 5-10 examples per class, markedly narrowing the gap between zero-shot and fully supervised models [29].

In parallel, contrastive-learned encoders (eg, MedCLIP) [30] and multimodal retrieval augmentation [31] can enrich representations and factual grounding, mitigating data scarcity and hallucination. In summary, improving MM-LLM image classification in medical domains is multifaceted, and combining hybrid ICL (image + text), contrastive pretraining, and retrieval augmentation offers a practical path to greater accuracy, robustness, and interpretability in cystoscopic diagnosis.

### Clinical Implications

MM-LLMs offer greater flexibility than task-specific endoscopy AI by combining visual recognition with contextual reasoning and narrative explanation. They can interpret morphology-diverse findings and integrate relevant clinical text, supporting a more context-aware understanding. Our results suggest potential applications in education and workflow support, including serving as virtual tutors for trainees and automating report generation to reduce workload and standardize documentation. However, their moderate diagnostic accuracy, particularly for rare or subtle lesions, limits their current use as autonomous diagnostic tools. Future efforts should focus on vision-conditioned ICL, multimodal retrieval-augmented training, and video-based modeling to enhance interpretive stability and diagnostic confidence [30,31]. Integration with patient-level data, cystoscopy-specific benchmark datasets, and human-in-the-loop oversight will be critical to ensure clinical safety and responsible implementation.

### Limitations

This study has several methodological strengths, including an unbiased and rigorous evaluation framework. The dual-task design enabled simultaneous assessment of reasoning transparency, adaptability, and accuracy—helping distinguish superficial pattern recognition from genuine clinical understanding. However, several limitations should be acknowledged. First, our ICL implementation was text-based only: models received brief written descriptions of tumor features without any paired visual exemplars. As a result, this study evaluated “text-based ICL” rather than full multimodal ICL, and the absence of a few-shot image or image-text examples likely constrained the models’ multimodal capabilities. The modest gains observed with ICL in our experiments may therefore underestimate the potential benefit of visual or hybrid (image + text) ICL. Future work should directly compare text-based, visual, and hybrid ICL strategies and explore complementary approaches such as contrastive-learned encoders and multimodal retrieval augmentation for cystoscopic diagnosis. Second, because the raw test images were drawn from heterogeneous sources, residual confounding from source-related differences and image-quality artifacts cannot be fully excluded. Although we applied a strict 3-layer quality control pipeline—image exclusion criteria, standardized preprocessing, and human verification of diagnostic utility—to mitigate these effects, some bias related to image source heterogeneity may remain. In addition, our evaluation relied on static images; incorporating temporal cues from cystoscopy videos may improve recognition of subtle or evolving lesions and reduce misclassification. Third, our dataset has an enriched abnormality prevalence (80.3%), substantially higher than that of typical clinical populations (20%-30%). Consequently, the reported positive predictive value and negative predictive value are inflated and not generalizable; they should be interpreted as dataset-specific rather than as estimates for real-world screening or hematuria-clinic settings.

### Conclusions

Using a clinically challenging, stress-test image set and a rigorous blinded evaluation framework, this study comprehensively assessed MM-LLMs for cystoscopic interpretation and lesion classification. Among the evaluated models, OpenAI-o3 demonstrated the most balanced and clinically coherent performance, followed by ChatGPT-4o and Gemini 2.5 Pro. These findings highlight the meaningful assistive potential of MM-LLMs in generating interpretable free-text rationales, supporting biopsy triage, and facilitating training. However, their performance in truly difficult differential diagnoses remains modest and requires further optimization before safe clinical integration.

## Data Availability

The datasets generated and/or analyzed during this study are available from the corresponding author upon reasonable request.

## Appendix

Multimedia Appendix 1 Additional methods, tables, and figures.

## Abbreviations

AI: artificial intelligence
CIS: carcinoma in situ
ICL: in-context learning
MCC: Matthews correlation coefficient
MM-LLM: multimodal large language model
Non-U Ca: non-urothelial carcinoma
NOTA: none of the above
pUC: papillary urothelial carcinoma
SOTA: state-of-the-art
Youden J: Youden J index
