# Supplementary material for: Multimodal Large Language Models for Cystoscopic Image Interpretation and Bladder Lesion Classification: Comparative Study
Source: J Med Internet Res. 2026 Jan 28;28:e87193. doi: 10.2196/87193 (PMC12895159; doi:10.2196/87193)
Supplement: Multimedia Appendix 1 [file jmir_v28i1e87193_app1.pdf]

MULTIMEDIA APPENDIX 1

Table of Contents

I. **Table S1:** Distribution of image source and results of memorization test

II. **Table S2:** Mean of pairwise score differences (column against row) among four MM-LLMs across four open questions.

III. **Table S3:** Inter-rater reliability by questions across models - fixed set, mean ICC value across three raters

IV. **Table S4:** Confusion matrices for biopsy-indication binary classification in the tumor-like lesion subset using zero-shot and in-context learning prompts.

V. **Table S5:** Confusion matrices for malignancy binary classification in the tumor-like lesion subset using zero-shot and in-context learning prompts

VI. **Table S6:** Performance of ChatGPT-4o in 7-class tumor-like lesion classification under zero-shot and in-context learning prompting

VII. **Table S7:** Performance of Gemini-2.5-Pro in 7-class tumor-like lesion classification under zero-shot and in-context learning prompting

VIII. **Table S8:** Performance of MedGemma-27B in 7-class tumor-like lesion classification under zero-shot and in-context learning prompting

IX. **Table S9**: Sensitivity analysis: Binary endpoint performance conditional on valid responses

X. **Supplementary Methods** (p11)

XI. **Figure S1:** Screenshot of the custom blinded-evaluation interface

Table S1: Distribution of image source and results of memorization test

	Image Sources	Counts	(%)	Memorization Test			
				OpenAI-o3	ChatGPT-4o	Gemini-2.5-Pro	MedGemma-27B
1	Industry Archives (Karl-Storz)	184	(45.9)	x	x	x	x
2	Reference Atlases	84	(20.9)	x	x	x	x
3	De-identified Clinical Websites	52	(13.0)	x	x	x	x
4	PubMed-indexed Repositories	36	(9.0)	x	x	x	x
5	Creative commons-licensed videos (Youtube)	45	(11.2)	x	x	x	x
	Total	401	(100)	Pass	Pass	Pass	Pass

The 401-image dataset comprised five source categories. To assess potential memorization from overlapping pretraining data, we performed two memorization tests for each model: (1) a **Textual Completion Test,** in which the model received only the opening segment of the original case description (without the image) and was asked to complete the description, provide a diagnosis, and name any source it "remembered"; and (2) a **Source Identification Test**, in which the model was shown the processed image and asked to identify the original source or provide a citation. We evaluated 100 images in total—60 from industry archives and atlas textbooks and 40 randomly sampled from de-identified clinical websites, PubMed-indexed repositories, and YouTube. None of the textual completions reproduced or closely matched the original descriptions, and no sources were correctly identified, indicating a very low risk of data leakage from model pretraining corpora. X: no memorization

Table S2: Mean of pairwise score differences (column against row) among four MM-LLMs across four open questions

Q1. Anatomic Site					
		Models			
		OpenAI-o3	ChatGPT-4o	Gemini-2.5-Pro	MedGemma-27B
Models	OpenAI-o3	–	-0.07	-0.03	-1.68 *
	ChatGPT-4o	0.07	–	0.05	-1.65 *
	Gemini-2.5-Pro	0.03	-0.05	–	-1.60 *
	MedGemma-27B	1.68 *	1.60 *	1.65 *	–

Q2. Findings					
		OpenAI-o3	ChatGPT-4o	Gemini-2.5-Pro	MedGemma-27B
Models	OpenAI-o3	–	-0.15 *	-0.27 *	-1.90 *
	ChatGPT-4o	0.15 *	–	-0.11	-1.74 *
	Gemini-2.5-Pro	0.27 *	0.11	–	-1.63 *
	MedGemma-27B	1.90 *	1.74 *	1.63 *	–

Q4. Lesion reasoning					
		OpenAI-o3	ChatGPT-4o	Gemini-2.5-Pro	MedGemma-27B
Models	OpenAI-o3	–	-0.06	-0.24 *	-1.32 *
	ChatGPT-4o	0.06	–	-0.19	-1.27 *
	Gemini-2.5-Pro	0.24 *	0.19	–	-1.08 *
	MedGemma-27B	1.32 *	1.27 *	1.08 *	–

Q5. Final Diagnosis					
		OpenAI-o3	ChatGPT-4o	Gemini-2.5-Pro	MedGemma-27B
Models	OpenAI-o3	–	-0.04	-0.19	-1.32 *
	ChatGPT-4o	0.04	–	-0.14	-1.27 *
	Gemini-2.5-Pro	0.19	0.14	–	-1.13 *
	MedGemma-27B	1.32 *	1.27 *	1.13 *	–

* Statistically significant at $q < 0.05$, where q = False Discovery Rate (FDR)-adjusted p-values

Table S3. Inter-rater reliability by question across models—fixed set, mean ICC value across three raters

		Models			
		OpenAI-o3	ChatGPT-4o	Gemini-2.5-Pro	MedGemma-27B
Whole test set (n=401)					
**Questions**					
Q1	Anatomic site	0.89	0.89	0.89	0.60
Q2	Findings	0.82	0.84	0.85	0.78
Q4	Lesion reasoning	0.93	0.92	0.92	0.89
Q5	Final diagnosis	0.94	0.92	0.93	0.88

ICC = Intraclass correlation coefficient

Intraclass correlation coefficients demonstrated excellent inter-rater reliability across both model and question domains. Among 16 ICC values, 14 ranged from 0.82 to 0.94, indicating high consistency among raters.

Table S4: Confusion matrices for biopsy-indication binary classification in the tumor-like lesion subset using zero-shot and in-context learning prompts.

Biopsy Indication											
		**Zero-shot prompt**						**ICL prompt**			
**OpenAI-o3**		Predicted						Predicted			
		No	Yes	Invalid	total			No	Yes	Invalid	total
Actual	No	23	17	–	40	Actual	No	27	13	–	40
Actual	Yes	12	60	1	73	Actual	Yes	14	59	–	73
	total	35	77	1	113		total	41	72	0	113
**ChatGPT-4o**											
		No	Yes	Invalid	total			No	Yes	Invalid	total
Actual	No	11	23	6	40	Actual	No	13	25	2	40
Actual	Yes	4	67	2	73	Actual	Yes	8	65	–	73
	total	15	90	8	113		total	21	90	2	113
**Gemini-2.5-Pro**											
		No	Yes	Invalid	total			No	Yes	Invalid	total
Actual	No	17	23	–	40	Actual	No	23	17	–	40
Actual	Yes	10	63	–	73	Actual	Yes	18	55	–	73
	total	27	86	0	113		total	41	72	0	113
**MedGemma-27B**											
		No	Yes	Invalid	total			No	Yes	Invalid	total
Actual	No	3	37	–	40	Actual	No	0	40	–	40
Actual	Yes	2	71	–	73	Actual	Yes	5	68	–	73
	total	5	108	0	113		total	5	108	0	113

Invalid: Model outputs failing to provide a single permissible choice

– : No Invalid outputs

Table S5: Confusion matrices for malignancy binary classification in the tumor-like lesion subset using zero-shot and in-context learning prompts

Presence of Malignancy

Zero-shot prompt

OpenAI-o3

		Predicted No	Predicted Yes	Predicted Invalid	total
Actual	No	28	31	–	59
Actual	Yes	10	43	1	54
	total	38	74	1	113

ChatGPT-4o

		No	Yes	Invalid	total
Actual	No	19	34	6	59
Actual	Yes	8	44	2	54
	total	27	78	8	113

Gemini-2.5-Pro

		No	Yes	Invalid	total
Actual	No	22	37	–	59
Actual	Yes	7	47	–	54
	total	29	84	0	113

MedGemma-27B

		No	Yes	Invalid	total
Actual	No	26	33	–	59
Actual	Yes	15	39	–	54
	total	41	72	0	113

ICL prompt

OpenAI-o3

		Predicted No	Predicted Yes	Predicted Invalid	total
Actual	No	34	25	–	59
Actual	Yes	16	38	–	54
	total	50	63	0	113

ChatGPT-4o

		No	Yes	Invalid	total
Actual	No	34	23	2	59
Actual	Yes	21	33	–	54
	total	55	56	2	113

Gemini-2.5-Pro

		No	Yes	Invalid	total
Actual	No	34	25	–	59
Actual	Yes	18	36	–	54
	total	52	61	0	113

MedGemma-27B

		No	Yes	Invalid	total
Actual	No	12	47	–	59
Actual	Yes	7	47	–	54
	total	19	94	0	113

Invalid: Model outputs failing to provide a single permissible choice

– : No Invalid outputs

Table S6: Performance of ChatGPT-4o in 7-class Tumor-like Lesion Classification under Zero-shot and In-context Learning Prompting (Strict Analysis)

Zero-shot		Predicted										Classification Metrics						
	**Confusion Matrix**	cystitis	polyps	papilloma	pUC	CIS	non-U Ca	NOTA	Invalid	total		Accuracy	Sensitivity	Specificity	PPV	NPV	Youden-J	MCC
	cystitis	2	0	0	2	10	0	0	4	18	cystitis	77.0	11.1	89.5	16.7	84.2	0.006	0.007
	polyps	0	1	3	2	1	0	0	0	7	polyps	94.7	14.3	100.0	100.0	94.6	0.143	0.368
	papilloma	0	0	3	8	1	0	0	0	12	papilloma	85.0	25.0	92.1	27.3	91.2	0.171	0.178
**Actual**	pUC	0	0	4	15	1	0	0	0	20	pUC	64.6	75.0	62.4	30.0	92.1	0.374	0.287
	CIS	4	0	0	0	12	0	0	1	17	CIS	81.4	70.6	83.3	42.9	94.1	0.539	0.447
	non-U Ca	0	0	0	16	0	0	0	1	17	non-U Ca	85.0	0.0	100.0	0.0	85.0	0.000	0.000
	NOTA	6	0	1	7	3	0	3	2	22	NOTA	83.2	13.6	100.0	100.0	82.7	0.136	0.336
	total	12	1	11	50	28	0	3	8	113	**AVG**	31.9	31.9	89.8	34.3	88.8	0.217	0.224

ICL prompt		Predicted										Classification Metrics						
	**Confusion Matrix**	cystitis	polyps	papilloma	pUC	CIS	non-U Ca	NOTA	Invalid	total		Accuracy	Sensitivity	Specificity	PPV	NPV	Youden-J	MCC
	cystitis	4	2	2	0	8	0	1	1	18	cystitis	75.2	22.2	85.3	22.2	85.3	0.075	0.075
	polyps	0	1	3	2	1	0	0	0	7	polyps	89.4	14.3	94.3	14.3	94.3	0.086	0.086
	papilloma	1	0	6	5	0	0	0	0	12	papilloma	76.1	50.0	79.2	22.2	93.0	0.292	0.211
**Actual**	pUC	0	0	8	11	1	0	0	0	20	pUC	75.2	55.0	79.6	36.7	89.2	0.346	0.299
	CIS	7	0	0	0	9	1	0	0	17	CIS	80.5	52.9	85.4	39.1	91.1	0.384	0.341
	non-U Ca	0	3	3	9	0	2	0	0	17	non-U Ca	85.8	11.8	99.0	66.7	86.4	0.107	0.238
	NOTA	6	1	5	3	4	0	2	1	22	NOTA	81.4	9.1	98.9	66.7	81.8	0.080	0.197
	total	18	7	27	30	23	3	3	2	113	**AVG**	31.0	31.0	88.8	31.5	88.5	0.198	0.199

pUC = papillary urothelial carcinoma, **CIS** = carcinoma in situ, **non-U Ca** = non-urothelial carcinoma, **NOTA** = none of the above, **ICL** = In-context Learning

PPV = Positive Predictive Value, **NPV** = Negative Predictive Value, **Youden-J**=Youden's J Index, **MCC**=Matthews Correlation Coefficient, **AVG** = micro-average

Invalid: Model outputs failing to provide a single permissible choice; Strict analysis: Invalid outputs were included in the denominator and treated as incorrect predictions.

Table S7: Performance of Gemini-2.5-Pro in 7-class Tumor-like Lesion Classification under Zero-shot and In-context Learning Prompting

**Zero-shot**		**Predicted**								**Classification Metrics**						
**Confusion Matrix**	cystitis	polyps	papilloma	pUC	CIS	non-U Ca	NOTA	total		Accuracy	Sensitivity	Specificity	PPV	NPV	Youden-J	MCC
**Actual** cystitis	6	0	0	1	9	0	2	18	cystitis	77.0	33.3	85.3	30.0	87.1	0.186	0.178
polyps	2	0	0	3	1	1	0	7	polyps	93.8	0	100	0	93.8	0	0
papilloma	1	0	2	5	2	2	0	12	papilloma	91.2	16.7	100	100	91.0	0.167	0.389
pUC	2	0	0	13	0	5	0	20	pUC	70.8	65.0	72.0	33.3	90.5	0.370	0.297
CIS	5	0	0	1	11	0	0	17	CIS	80.5	64.7	83.3	40.7	93.0	0.480	0.403
non-U Ca	0	0	0	12	1	4	0	17	non-U Ca	76.1	23.5	85.4	22.2	86.3	0.090	0.087
NOTA	4	0	0	4	3	6	5	22	NOTA	83.2	22.7	97.8	71.4	84.0	0.205	0.337
total	20	0	2	39	27	18	7	113	**AVG**	36.3	36.3	89.4	36.3	89.4	0.257	0.257

**ICL prompt**		**Predicted**								**Classification Metrics**						
**Confusion Matrix**	cystitis	polyps	papilloma	pUC	CIS	non-U Ca	NOTA	total		Accuracy	Sensitivity	Specificity	PPV	NPV	Youden-J	MCC
**Actual** cystitis	10	1	0	1	6	0	0	18	cystitis	70.8	55.6	73.7	28.6	89.7	0.292	0.231
polyps	2	0	2	1	1	1	0	7	polyps	90.3	0	96.2	0	93.6	-0.038	-0.049
papilloma	2	0	3	4	2	1	0	12	papilloma	88.5	25.0	96.0	42.9	91.5	0.210	0.269
pUC	4	0	2	9	0	5	0	20	pUC	72.6	45.0	78.5	31.0	86.9	0.235	0.205
CIS	9	0	0	1	7	0	0	17	CIS	82.3	41.2	89.6	41.2	89.6	0.308	0.308
non-U Ca	1	2	0	9	1	4	0	17	non-U Ca	78.8	23.5	88.5	26.7	86.7	0.121	0.127
NOTA	7	1	0	4	0	4	6	22	NOTA	85.8	27.3	100	100	85.1	0.273	0.482
total	35	4	7	29	17	15	6	113	**AVG**	34.5	34.5	89.1	34.5	89.1	0.236	0.236

pUC = papillary urothelial carcinoma, **CIS** = carcinoma in situ, **non-U Ca** = non-urothelial carcinoma, **NOTA** = none of the above, **ICL** = In-context Learning

PPV = Positive Predictive Value, **NPV** = Negative Predictive Value, **Youden-J**=Youden's J Index, **MCC**=Matthews Correlation Coefficient, **AVG** = micro-average

Table S8: Performance of MedGemma-27B in 7-class Tumor-like Lesion Classification under Zero-shot and In-context Learning Prompting

**Zero-shot**		Predicted								Classification Metrics						
**Confusion Matrix**		cystitis	polyps	papilloma	pUC	CIS	non-U Ca	NOTA	total		Accuracy	Sensitivity	Specificity	PPV	NPV	Youden-J	MCC
**Actual**	cystitis	0	2	3	11	2	0	0	18	cystitis	83.2	0	99.0	0	83.9	-0.011	-0.041
	polyps	0	0	3	4	0	0	0	7	polyps	90.3	0	96.2	0	93.6	-0.038	-0.049
	papilloma	0	0	7	5	0	0	0	12	papilloma	73.5	58.3	75.3	21.9	93.8	0.336	0.230
	pUC	0	0	7	13	0	0	0	20	pUC	51.3	65.0	48.4	21.3	86.5	0.134	0.103
	CIS	1	0	3	3	9	0	1	17	CIS	91.2	52.9	97.9	81.8	92.2	0.509	0.613
	non-U Ca	0	1	2	14	0	0	0	17	non-U Ca	85.0	0	100	0	85.0	0	0
	NOTA	0	1	7	11	0	0	3	22	NOTA	82.3	13.6	98.9	75.0	82.6	0.125	0.269
	total	1	4	32	61	11	0	4	113	**AVG**	28.3	28.3	88.1	28.3	88.1	0.164	0.164

**ICL prompt**		Predicted								Classification Metrics						
**Confusion Matrix**		cystitis	polyps	papilloma	UC	CIS	non-U Ca	NOTA	total		Accuracy	Sensitivity	Specificity	PPV	NPV	Youden-J	MCC
**Actual**	cystitis	0	0	1	16	1	0	0	18	cystitis	80.5	0	95.8	0	83.5	-0.042	-0.083
	polyps	0	1	1	5	0	0	0	7	polyps	92.9	14.3	98.1	33.3	94.6	0.124	0.186
	papilloma	1	0	4	6	1	0	0	12	papilloma	86.7	33.3	93.1	36.4	92.2	0.264	0.274
	pUC	0	0	0	20	0	0	0	20	pUC	41.6	100	29.0	23.3	100	0.290	0.260
	CIS	3	1	1	5	6	0	1	17	CIS	88.5	35.3	97.9	75.0	89.5	0.332	0.463
	non-U Ca	0	0	1	16	0	0	0	17	non-U Ca	85.0	0	100	0	85.0	0	0
	NOTA	0	1	3	18	0	0	0	22	NOTA	79.7	0	98.9	0	80.4	-0.011	-0.047
	total	4	3	11	86	8	0	1	113	**AVG**	27.4	27.4	87.9	27.4	87.9	0.153	0.153

pUC = papillary urothelial carcinoma, **CIS** = carcinoma in situ, **non-U Ca** = non-urothelial carcinoma, **NOTA** = none of the above, **ICL** = In-context Learning

PPV = Positive Predictive Value, **NPV** = Negative Predictive Value, **Youden-J**=Youden's J Index, **MCC**=Matthews Correlation Coefficient, **AVG** = micro-average

Table S9. Sensitivity Analysis: Binary Endpoint Performance Conditional on Valid Responses

Tasks	MM-LLMs	Valid response rate (%)	Classification Metrics						
			Accuracy (%)	Sensitivity (%)	Specificity (%)	PPV (%)	NPV (%)	Youden-J	MCC
**Tumor-like Lesion Subset (n = 113)**									
**Biopsy Indication (Yes / No)**									
Zero-shot prompting									
	OpenAI-o3	99.1	74.1	83.3	57.5	77.8	65.7	0.408	0.422
	ChatGPT-4o	92.9	74.3	94.8	32.4	74.4	73.3	0.267	0.357
	Gemini-2.5-Pro	100.0	70.8	86.3	42.5	73.3	63	0.288	0.323
	MedGemma-27B	100.0	65.5	97.3	7.5	65.7	60	0.048	0.111
In-context learning									
	OpenAI-o3	100.0	76.1	80.8	67.5	81.9	65.6	0.483	0.481
	ChatGPT-4o	98.2	70.3	89	34.2	72.2	61.9	0.232	0.282
	Gemini-2.5-Pro	100.0	69	75.3	57.5	76.4	56.1	0.328	0.327
	MedGemma-27B	100.0	60.2	93.2	0	63	0	-0.069	-0.159
**Presence of Malignancy (Yes / No)**									
Zero-shot prompting									
	OpenAI-o3	99.1	63.4	81.1	47.5	58.1	73.7	0.286	0.302
	ChatGPT-4o	92.9	60	84.6	35.9	56.4	70.4	0.205	0.234
	Gemini-2.5-Pro	100.0	61.1	87	37.3	56	75.9	0.243	0.278
	MedGemma-27B	100.0	57.5	72.2	44.1	54.2	63.4	0.163	0.169
In-context learning									
	OpenAI-o3	100.0	63.7	70.4	57.6	60.3	68	0.280	0.282
	ChatGPT-4o	98.2	60.4	61.1	59.7	58.9	61.8	0.208	0.208
	Gemini-2.5-Pro	100.0	62	66.7	57.6	59	65.4	0.243	0.244
	MedGemma-27B	100.0	52.2	87	20.3	50	63.2	0.074	0.099

PPV = Positive PredictiveValue; NPV = Negative Predictive Value; Youden-J = Youden's J Index; MCC=Matthews Correlation Coefficient

Valid response rate = (Total - Invalid) / Total. Invalid denotes outputs failing to provide a single permissible choice

Conditional-on-valid analysis: Metrics were calculated among valid model outputs only; invalid outputs were excluded from the denominator.

X. Supplementary Methods

5. Prompt design

5.1 Prompt design with open-ended questions for image interpretation

We used a role-based, zero-shot prompt tailored to cystoscopy. The prompt primed domain reasoning (“Suppose you are a urologist”) and briefly contextualized the procedure, followed by stepwise instructions to encourage explicit intermediate reasoning. The query comprised five domains: (1) anatomic site (free text), (2) endoscopic findings (free text), (3) presence or absence of a pathological lesion (binary), (4) lesion diagnostic reasoning and justification if present (free text), and (5) final diagnosis (free text). Wording and answer structure were identical across models and evaluation rounds to preserve comparability.

Exact prompt:
Suppose you are a urologist. This is a photo captured during the cystoscopy procedure. Cystoscopy is a procedure that uses a cystoscope to look inside the urethra and bladder. Let’s think step by step. Please answer the following questions based on the image provided:
1. What is the anatomic site of the urinary tract in the image?
2. What's your findings from this image?
3. Is there any pathological lesion? Answer = present(1) , absent(0)
4. If present, what is the lesion and the explanation?
5. What is the final diagnosis for the testee?

5.2 Prompt design for tumor-like lesion classification task with multiple-choice diagnostic framework

We compared two prompting strategies for cystoscopic diagnosis of tumor-like lesions: zero-shot and in-context learning (ICL). Both adopted a role-based setup (“Suppose you are a urologist”) and constrained the task to image-only visual cues. The zero-shot prompt presented a single forced-choice label set; the ICL prompt prepended a small support set of labeled examples written in the same schema, followed by the query. Models were instructed to output a single best diagnosis from the provided options and a brief rationale grounded in endoscopic morphology.

Zero-shot prompt:
Suppose you are a urologist. This is a photo captured during the cystoscopy procedure. Cystoscopy is a procedure that uses a cystoscope to look inside the urethra and bladder.
Based only on the cystoscopic appearance, what is the most likely diagnosis?
1. Cystitis
2. Polyps
3. Papilloma
4. Urothelial Carcinoma
5. Carcinoma in situ
6. Non-Urothelial malignant tumor (eg. squamous cell carcinoma/adenocarcinoma etc,.)
7. None of the above

In Context Learning (ICL) prompt:
For your reference:
===
Cystoscopic features of six tumor-like lesions:
Cystitis
- Mucosal hyperemia, confluent vessels, edema, petechiae, friability, hemorrhage
- Turbid urine
- Cystitis cystica: pearly white, translucent cysts along urothelium
Polyps
- Benign-appearing mucosal protrusion
- Plump mucosa, sometimes with visible delicate vessels
Papilloma
- Villous, papillary tumor-like lesion
- Delicate mucosal villi, frond-like projections
Urothelial Carcinoma
- Variable appearance: papillary (seaweed/cauliflower-like, pedunculated or sessile) or sessile masses
- May have vascular core, mucosal irregularity, color variation
- Sessile forms often higher grade
Carcinoma in situ
- Flat erythematous, velvety or granular patches with sharp borders
- Lacks exophytic or papillary growth
- Can be focal, multifocal, or diffuse
Non-Urothelial Malignant Tumor (e.g., squamous cell carcinoma, adenocarcinoma)
- Sessile or ulceroinfiltrative masses, not papillary
- Morphology distinct from urothelial carcinoma
None of the above
- Image shows findings not consistent with cystitis, polyps, papilloma, urothelial carcinoma, carcinoma in situ, or non-urothelial malignant tumors.
===
Suppose you are a urologist. This is a photo captured during the cystoscopy procedure. Cystoscopy is a procedure that uses a cystoscope to look inside the urethra

and bladder.
Based only on the cystoscopic appearance, what is the most likely diagnosis?
1. Cystitis
2. Polyps
3. Papilloma
4. Urothelial Carcinoma
5. Carcinoma in situ
6. Non-Urothelial malignant tumor (eg. squamous cell carcinoma/adenocarcinoma etc,.)
7. None of the above

Figure S1: Screenshot of the custom blinded-evaluation interface

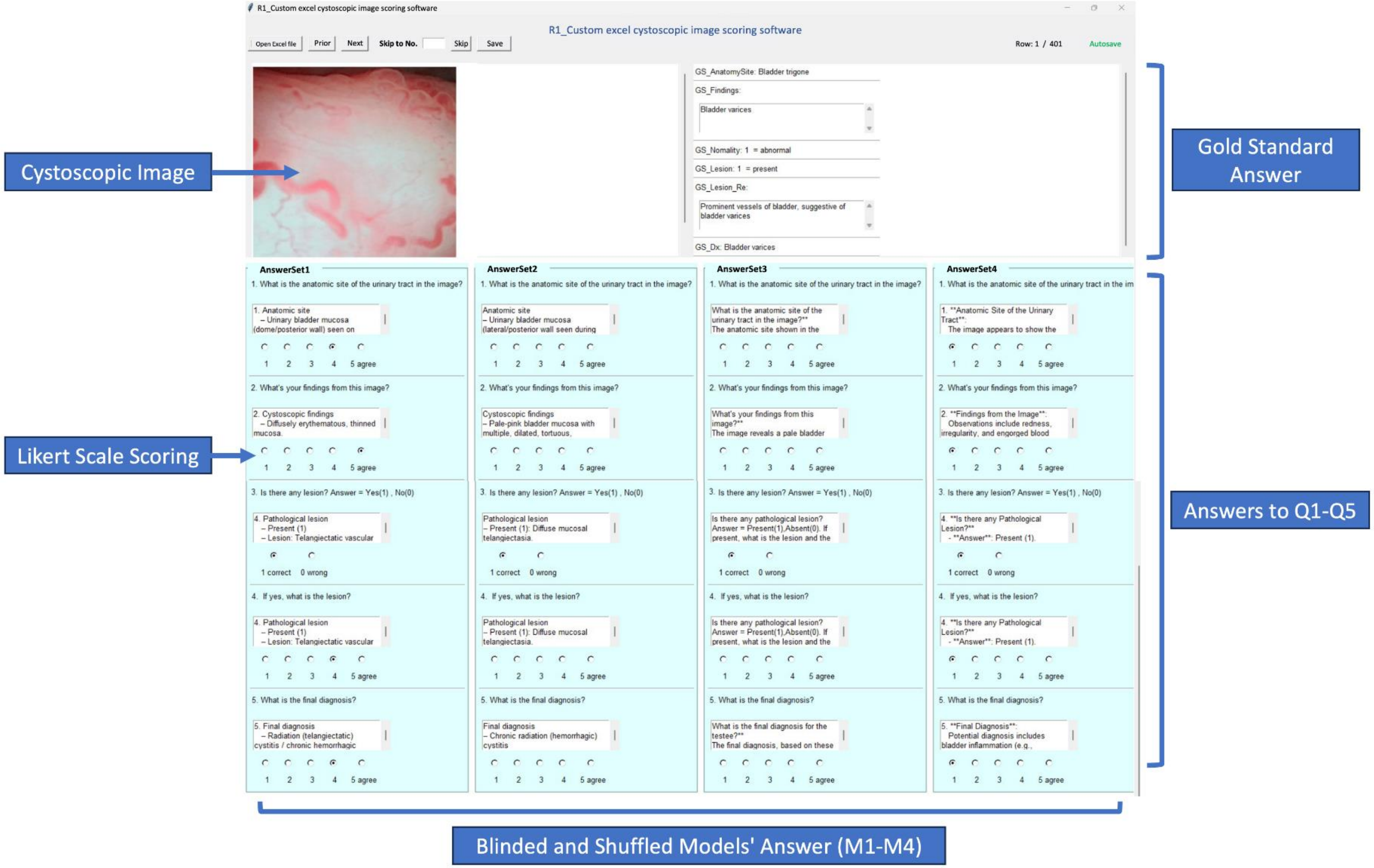

Screenshot of the custom rater-blinded, model-anonymized evaluation interface. The left panel shows a single cystoscopic image. The right panel displays the gold-standard reference answers (anatomic site, findings, binary lesion detection, lesion reasoning, and final diagnosis). The lower panes (AnswerSet1–4) present anonymized, randomly ordered outputs from models M1–M4 (answers to Q1–Q5). Raters score each model's answers against the reference using a 5-point Likert scale (1 = disagree to 5 = agree). Blue callouts highlight the key interface elements and their layout within the blinded workspace.
